# Improving carbon monoxide tolerance of *Cupriavidus necator* H16 through adaptive laboratory evolution

**DOI:** 10.3389/fbioe.2023.1178536

**Published:** 2023-04-24

**Authors:** Charles Wickham-Smith, Naglis Malys, Klaus Winzer

**Affiliations:** BBSRC/EPSCR Synthetic Biology Research Centre (SBRC), School of Life Sciences, Biodiscovery Institute, University of Nottingham, Nottingham, United Kingdom

**Keywords:** adaptive laboratory evolution (ALE), *Cupriavidus necator*, *Ralstonia eutropha*, carbon monoxide tolerance, cytochrome *bd* ubiquinol oxidase, [NiFe]-hydrogenase, syngas, gas fermentation

## Abstract

**Background:** The toxic gas carbon monoxide (CO) is abundantly present in synthesis gas (syngas) and certain industrial waste gases that can serve as feedstocks for the biological production of industrially significant chemicals and fuels. For efficient bacterial growth to occur, and to increase productivity and titres, a high resistance to the gas is required. The aerobic bacterium *Cupriavidus necator* H16 can grow on CO_2_ + H_2_, although it cannot utilise CO as a source of carbon and energy. This study aimed to increase its CO resistance through adaptive laboratory evolution.

**Results:** To increase the tolerance of *C. necator* to CO, the organism was continually subcultured in the presence of CO both heterotrophically and autotrophically. Ten individual cultures were evolved heterotrophically with fructose in this manner and eventually displayed a clear growth advantage over the wild type strain. Next-generation sequencing revealed several mutations, including a single point mutation upstream of a cytochrome *bd* ubiquinol oxidase operon (*cydA2B2*), which was present in all evolved isolates. When a subset of these mutations was engineered into the parental H16 strain, only the *cydA2B2* upstream mutation enabled faster growth in the presence of CO. Expression analysis, mutation, overexpression and complementation suggested that *cydA2B2* transcription is upregulated in the evolved isolates, resulting in increased CO tolerance under heterotrophic but not autotrophic conditions. However, through subculturing on a syngas-like mixture with increasing CO concentrations, *C. necator* could also be evolved to tolerate high CO concentrations under autotrophic conditions. A mutation in the gene for the soluble [NiFe]-hydrogenase subunit *hoxH* was identified in the evolved isolates. When the resulting amino acid change was engineered into the parental strain, autotrophic CO resistance was conferred. A strain constitutively expressing *cydA2B2* and the mutated *hoxH* gene exhibited high CO tolerance under both heterotrophic and autotrophic conditions.

**Conclusion:** *C. necator* was evolved to tolerate high concentrations of CO, a phenomenon which was dependent on the terminal respiratory cytochrome *bd* ubiquinol oxidase when grown heterotrophically and the soluble [NiFe]-hydrogenase when grown autotrophically. A strain exhibiting high tolerance under both conditions was created and presents a promising chassis for syngas-based bioproduction processes.

## Introduction

Rising concerns over significant environmental issues and the global over-reliance on fossil fuels has led to increased interest in the recycling of C1 gases and the use of renewable chemicals and biofuels (IPCC, 2018). Developing and implementing sustainable strategies to redirect C1 gases from their source as a feedstock for biotechnological applications may be an important strategy to reduce greenhouse gas emissions in the future. *Cupriavidus necator* H16 is a particularly well-suited organism for C1 fermentation due to its ability to grow on CO_2_ + H_2_ to produce valuable bioproducts, including polymers and potentially chemicals and fuels (Müller et al., 2013; Lu and Yu, 2017; Windhorst and Grescher, 2019).

Synthesis gas (syngas) is a promising energy rich feedstock for microbial fermentation (Bengelsdorf et al., 2013; Takors et al., 2018), which could be utilised by *C. necator* to produce these and other bioproducts. However, in addition to varying amounts of CO_2_ and H_2_, syngas contains high concentrations of CO, which the wild type is unable to metabolise (Heinrich et al., 2018). *C. necator* can be genetically engineered to oxidise CO to CO_2_ by introducing and expressing a functional carbon monoxide dehydrogenase (CODH), which under specific conditions enhanced PHB synthesis (Heinrich et al., 2018). There have also been claims that CO oxidation was achieved by anchoring an *Escherichia coli*-produced anaerobic CODH from *Acetobacterium woodii* on its cell surface (Hyeon et al., 2015). The [NiFe] hydrogenases of *C. necator* show low affinity towards CO and appear to be unaffected by its presence (Schneider et al., 1979; Burgdorf et al., 2005; Vincent et al., 2005). Additionally, the cytochromes of *C. necator* show low affinity for CO (Bernard et al., 1974). Despite this, at high concentrations (90%), CO has been shown to drastically inhibit the growth and respiration of *C. necator* (Cypionka and Meyer, 1982). Syngas typically contains 30%–60% CO (Higman and van der Burgt, 2008) a range in which *C. necator* growth is likely to be significantly inhibited.

Numerous bacteria are sensitive to CO, their growth and respiration becoming inhibited in the presence of the gas (Cypionka and Meyer, 1982; King, 2003; Desmard et al., 2009; Nobre et al., 2009; Wareham et al., 2016). Aerobic CO oxidisers belonging to the genera *Burkholderia, Mesorhizobium* and *Stenotrophomonas* are inhibited by and are unable to adapt to CO concentrations higher than 1% following incubations lasting several months (King, 2003). However, other aerobic CO oxidisers can grow with higher CO concentrations including *Xanthobacter* sp. strain INA43/2-2 (20%) and *Bradyrhizobium japoncium* USDA 110 (50%) (Lorite et al., 2000; King, 2003). *Oligotropha carboxidovorans* was found to tolerate 90% CO, although with reduced growth (Cypionka and Meyer, 1982). *Mycobacterium smegmatis* grows well in the presence of 30% CO and appears to adapt by remodelling its respiratory chain (Bayly et al., 2021). Certain anaerobic CO utilising bacteria (carboxydotrophs) can tolerate the gas at high concentrations and use it for growth and production of industrially relevant products such as ethanol, butyrate, acetate, butanol and methane (Vega et al., 1989; Grethlein et al., 1991; Luo et al., 2013). Recently, industrial production of acetone and isopropanol from syngas has been achieved using *Clostridium autoethanogenum* with high CO concentrations (Liew et al., 2022). Furthermore, high tolerance to CO has been documented in carboxydotrophs such as the acetogen *Butyribacterium methylotrophicum* (Lynd et al., 1982) and in various species of *Carboxydocella* (Sokolova et al., 2004; Slepova et al., 2006). Adaptation to growth in the presence of CO has been observed in several different carboxydotrophs, a process which can take a period of weeks to months (Lynd et al., 1982; O’Brien et al., 1984; Oelgeschläger and Rother, 2008; Esquivel-Elizondo et al., 2017). *B. methylotrophicum* growth is completely inhibited in presence of 16% CO but following adaptation by successive transfer with increasing concentrations of the gas, the evolved strain was able to grow with CO due to the altered regulation of ferredoxin-NAD oxidoreductase (Shen et al., 1999). The mechanisms underlying bacterial CO resistance are however still not well understood.

For *C. necator* to be efficiently cultured with syngas or any alternative feedstock containing high concentrations of CO, adaptations to cope with its toxic effects are essential. Industrial organisms are typically produced through adaptive laboratory evolution (ALE) (Debabov, 2015); whether tolerance of *C. necator* to CO can be improved through this process has yet to be investigated. This study explored ALE in the presence of CO, with a view to produce genetically adapted *C. necator* strains displaying a growth advantage in the presence of high CO concentrations and to establish and exploit the underlying genetic changes.

## Materials and methods

### Bacterial strains, media and growth conditions

All species and strains used in this study are listed in Supplementary Table S1 together with their relevant genotypic or phenotypic features. Lysogeny broth (LB) was used for general maintenance of *E. coli* and *C. necator* H16 derivatives. Low-salt-LB (LSLB)-MOPS medium (Lenz et al., 1994) was used when growing *C. necator* H16 as recipient in conjugative procedures. Chemically defined medium (MM) based on Schlegel et al. (1961) including modified trace element solution SL7 (Trüper and Pfennig, 1981) was used for *C. necator* H16 growth assays and was supplemented with either 0.4% fructose (F-MM; heterotrophic growth) or CO_2_/H_2_ mixtures as indicated for the individual experiments in the headspace (autotrophic growth). The MM contained (per litre) 9 g sodium phosphate, 1.5 g potassium phosphate, 1.0 g ammonium chloride, 0.2 g magnesium sulphate, 0.02 g calcium chloride, 1.2 mg iron (III) citrate and 1 mL SL7 solution [hydrochloric acid (25%, v/v) 1 mL/L, zinc chloride 70 mg/L, manganese (II) chloride 100 mg/L, boric acid 60 mg/L, cobalt (II) chloride 200 mg/L, copper (II) chloride 20 mg/L, nickel (II) chloride 20 mg/L, sodium molybdate 40 mg/L], with the addition of 15 g/L agar (Bacto-agar, BD) if for solid agar medium. The pH value was adjusted to pH 6.9 using 1 M HCl. For counter selection during conjugation with *E. coli* S17-1, 10 μg/mL gentamicin was added to agar plates.

*E. coli* was grown aerobically in LB at 37°C and liquid cultures shaking at 200 revolutions per minute (RPM) in 50 mL Falcon tubes. *C. necator* was grown in LB or MM at 30°C and liquid cultures shaking at 200 revolutions per minute (RPM) in 50 mL Falcon tubes.

For growth under a defined headspace, *C. necator* was cultured in serum bottles, which were sealed with butyl rubber stoppers and aluminium clamps. Under these conditions, the respective culture and serum bottle volumes were 20 mL F-MM and 150 mL, respectively, for heterotrophic growth, and 25 mL MM and 250 mL, respectively, for autotrophic growth. Different volumes of CO, N_2_, CO_2_ and H_2_ were injected into the serum bottles through a hypodermic needle and 0.2 µm nitrocellulose filter using a commercial gas exchange system (GR Instruments). Gas mixtures were injected in addition to the existing 1 bar atmospheric pressure to give a total of 2 bars (heterotrophic growth) or 2.4 bars (autotrophic growth).

For heterotrophic growth comparisons, three colonies of each strain were individually grown in 5 mL F-MM to mid exponential phase. These were then subcultured in 20 mL F-MM in serum bottles with either 50% CO/50% air (v/v) or 50% N_2_/50% air (v/v). Heterotrophic growth under a CO atmosphere was initiated with a starting OD_600_ of 0.05 and heterotrophic growth under an N_2_ atmosphere was initiated with a starting OD_600_ of 0.02. For autotrophic growth, strains were first cultured in 5 mL F-MM to mid exponential phase, then washed with PBS. Autotrophic growth comparisons were performed with headspaces containing either 15% CO/65% H_2_/10% CO_2_/10% air or 30% CO/50% H_2_/10% CO_2_/10% air or 50% CO/30% H_2_/10% CO_2_/10% air. Control growth comparisons were performed with equivalent headspaces where CO had been replaced with N_2_, i.e., either 15% N_2_/65% H_2_/10% CO_2_/10% air or 30% N_2_/50% H_2_/10% CO_2_/10% air or 50% N_2_/30% H_2_/10% CO_2_/10% air. All autotrophic experiments were started using an OD_600_ of 0.01.

### DNA isolation, PCR, cloning and transformation

Plasmid DNA was isolated using the Qiagen Plasmid Miniprep Kit and genomic DNA was extracted using the GenElute Bacterial Genomic DNA Kit (Sigma-Aldrich). Oligonucleotide primers from Sigma-Alrich were used for PCR amplifications, performed using either the DreamTaq PCR Master Mix (×2) (Thermofisher Scientific) or Q5 polymerase (New England Biolabs). The former was used for screening of clones by colony PCR whereas Q5 polymerase was used for amplification of sequences for cloning. Restriction enzymes were purchased from NEB and “FastDigest” enzymes from Fermentas (Thermofisher Scientific). The Qiagen DNA Gel Extraction Kit was used to extract gel purified DNA for subsequent cloning. Chemically competent *E. coli* were prepared and transformed by heat shock as previously described (Sambrook et al., 1989). Plasmids used or generated in this study are listed in Supplementary Table S2 and oligonucleotides are listed in Supplementary Table S3.

### Construction of knock-out/knock-in plasmids

All plasmids were constructed using NEBuilder HiFi DNA assembly (New England Biolabs) following the manufacturer’s instructions. Derivatives of pLO3 (Lenz and Friedrich, 1998) were used to carry out deletions and integrations in *C. necator* H16 as previously described (Arenas-López et al., 2019). The tetracycline resistance marker and the *sacB* gene of pLO3 allow for counter-selection in the presence of sucrose. Homology arms (750-850 bp in length) upstream and downstream of the six deletion sites were PCR-amplified from the *C. necator* genome with primers containing overlapping regions (Supplementary Table S3). The left homology arm (LHA) forward primer contained base pairs overlapping with pLO3 at the chosen restriction site and the LHA reverse primer contained base pairs overlapping with right homology arm (RHA) forward primer. The RHA forward primer contained base pairs overlapping with the LHA reverse primer and the RHA reverse primer contained base pairs overlapping with pLO3 at the second restriction site. The LHA and RHA PCR fragments could therefore be used in a HiFi-DNA assembly reaction with restriction-digested pLO3, to construct the respective knock-out plasmid. The LHA reverse and RHA forward primers for all knock-in plasmids contained the altered single nucleotide or deletion to generate the desired mutation but otherwise covered the entire native sequence.

### Adaptive laboratory evolution of *C. necator* H16

For the heterotrophic evolution of CO-tolerant *C. necator* H16, a single colony was used to inoculate 5 mL F-MM and grown to an OD_600_ of around 2. This culture was used to inoculate ten separate 150 mL serum bottles containing 20 mL F-MM under a 2 bar atm of 50% CO/50% air (v/v). These cultures were shaken at 200 RPM in a 30°C incubator for at least 48 h to reach an OD_600_ of around 2. This culture was used to inoculate 20 mL F-MM to an OD_600_ of 0.01 in a fresh serum bottle and this was incubated under the same conditions. This subculturing cycle was repeated until cultures showed a clear improvement in growth. The cultures were monitored 18 h after each inoculation to observe whether the OD_600_ had dramatically increased. After this length of time wild type cultures would typically yield an OD_600_ of around 0.1, whereas evolved cultures yielded an OD_600_ of around 1, which was visually distinguishable. The purity of these cultures was checked taking a 1 mL sample of OD_600_ cells and making serial dilutions, plating out 10^−5^ on F-MM agar plates. Single colonies were screened by colony PCR using primers for the *hsdRN* gene (H16_A0006) to ensure they did represent H16 strain derivatives. Samples were taken every three subcultures and preserved in case they needed reanalysing or as a backup if cultures became contaminated. Single colonies of CO tolerant evolved cultures where than obtained by plating. To evolve the H16 strain under autotrophic conditions, a similar growth regime was used. A single H16 colony was grown in 5 mL F-MM pre-culture, then diluted to an OD_600_ of 0.01 in 250 mL serum bottles containing 25 mL MM and a headspace of 15% CO/65% H_2_/10% CO_2_/10% air (v/v) under 2.4 bar pressure. Following 18 days of incubation at 200 RPM in a 30°C incubator, cultures were used to reinoculate fresh serum bottles to an OD_600_ of 0.01 with higher CO headspace concentrations of 30% and 50%, respectively (represented by gas mixtures consisting of 30% CO/50% H2/10% CO2/10% air (v/v) and 50% CO/30% H2/10% CO2/10% air (v/v), respectively, under 2.4 bar pressure). The subculturing cycle was then repeated under the 50% CO atmosphere until cultures took only 3 days to reach stationary phase. Single colonies of CO tolerant evolved cultures were then obtained by plating.

### Measurement of *rfp* reporter gene expression

Promoter activity was measured using derivatives of the red fluorescence protein (mRFP1)-encoding biosensor plasmid pEH006 (Hanko et al., 2017) and an Infinite^®^ M1000 PRO micro plate reader (Tecan, Switzerland). 100 μL of sample was pipetted into a well of a black 96-well plate (Greiner Bio One International, Germany; Cat. No 655090). Fluorescence was measured using 585 nm as excitation wavelength and an emission wavelength of 620 nm. The gain factor for fluorescence was set manually to 80%. The OD_600_ of each sample was measured using the micro plate reader to normalise fluorescence by optical density. To correct for background fluorescence, the normalised fluorescence of the empty vector control (*C. necator* pEH006E; Hanko et al., 2017) was subtracted from each sample.

### Raw data for whole genome sequencing analysis

All raw NGS data used in this study is deposited in the NCBI Sequence Read Archive (SRA; https://www.ncbi.nlm.nih.gov/sra) and can be found under BioProject ID: PRJNA945825. The accession numbers for the strains used or created in this study are as follows: parental H16 wild type, SRX19749121; EA, SRX19746595; EB, SRX19746596; EC, SRX19746603; E1, SRX19746604; E2, SRX19746605; E3, SRX19746606; E4, SRX19746607; E5, SRX19746608; E6, SRX19746609; E7, SRX19746610; E8, SRX19746597; E9, SRX19746598; E10, SRX19746599; SynE1, SRX19746600; SynE2, SRX19746601; SynE3, SRX19746602.

### Genome sequencing analysis and SNP calling

Sequencing of genomic DNA was performed at MicrobesNG (University of Birmingham, United Kingdom), where their in-house software was used to trim sequencing reads and assess their quality. The “Standard Whole Genome Service” included Illumina next-generation sequencing with a guaranteed minimum coverage of 30x. The published genomes of *C. necator* H16 (Pohlmann et al. 2006; Little et al., 2019) was used as a template to map paired end reads to by using the software CLC Genomics work bench 22 (Qiagen). The following parameters set by the read mapping analysis were used: Masking mode = no masking; Mismatch cost = 2; Cost of insertions and deletions = linear gap cost; Insertion cost = 3; Deletion cost = 3; Insertion open cost = 6; Insertion extend cost = 1; Deletion open cost = 6; Deletion extend cost = 6; Length fraction = 0.5; Similarity fraction = 0.8; Global alignment = No; Auto-detect paired distances = yes; Non-specific match handling = map randomly.

The mapped reads were used to call single nucleotide polymorphisms (SNPs) by using CLC Genomics Workbench 22 (Qiagen). The following parameters were used for the Mapping and Variants workflow: Ploidy = 1; Ignore positions with coverage above = 100,000; Restrict calling to target regions = not set; Ignore broken pairs = yes; Ignore non-specific matches = reads; Minimum coverage = 10; Minimum count = 2; Minimum frequency (%) = 80.0; Base quality filter = yes; Neighbourhood radius = 5; Minimum central quality = 20; Minimum neighbourhood quality = 15; Read direction filter = no; Relative read direction filter = yes; Significance (%) = 1.0; Read position filter = no; Remove pyro-error variants = yes; In homopolymer regions with minimum length = 5; With frequency below = 0.7.

### Statistical analysis

All data are presented as mean ± standard deviation (SD) with the number of independent replicas given in the respective figure legends. Statistical analyses were performed using GraphPad Prism software version 9 (GraphPad, La Jolla, CA, United States), employing a paired *t*-test for side-by-side comparisons of the wild type and a given mutant under a particular condition, or for side-by-side comparisons of the same strain under two different conditions, with *p* values below 0.05 considered to be statistically significant.

## Results

### Carbon monoxide tolerance of *C. necator* H16

When grown in a complex medium with pyruvate, *C. necator* H16 is significantly inhibited by high concentrations (90%) of CO (Cypionka and Meyer, 1982), however the extent to which the organism is inhibited by lower concentrations of CO has not been fully investigated. To determine the inhibitory effect CO has on growth, *C. necator* H16 was grown both heterotrophically with fructose and autotrophically with CO_2_ + H_2_ in the presence of increasing concentrations of CO (0%, 15%, 30%, 50% v/v) together with air in the headspace.

**FIGURE 1:**
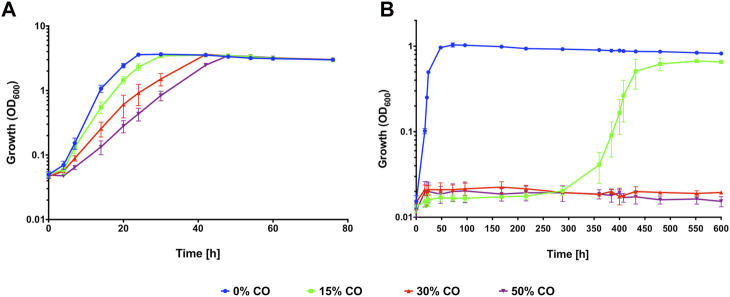
*C. necator* H16 heterotrophic growth **(A)** or autotrophic growth **(B)** with increasing concentrations of CO. *C. necator* was grown at 30°C in **(A)** 20 mL F-MM contained in 150 mL serum bottles under a 2 bar atm of 50% N_2_/50% air (v/v) (blue circles), 15% CO/35% N_2_/50% air (v/v) (green squares), 30% CO/20% N_2_/50% air (v/v) (red triangles) and 50% CO/50% air (v/v) (purple inverted triangles), respectively and in **(B)** 25 mL MM contained in 250 mL serum bottles under a 2.4 bar atm of 0% CO/80% H_2_/10% CO_2_/10% air (v/v) (blue circles); 15% CO/65% H_2_/10% CO_2_/10% air (v/v) (green squares); 30% CO/50% H_2_/10% CO_2_/10% air (v/v) (red triangles) and 50% CO/30% H_2_/10% CO_2_/10% air (v/v) (purple inverted triangles), respectively. Error bars represent the standard deviation of the mean for three independent replicates.

For growth on fructose, 50% (v/v) was chosen as the maximum CO concentration as it is within the range typically observed for syngas mixtures and because increasing above this threshold would have severely reduced the volume of air (and thus oxygen) available in the headspace, with further negative effects on *C. necator* growth. Pre-cultures were grown in fructose minimal medium (F-MM) and used to inoculate serum bottles containing F-MM and headspaces comprised of 50% air with different CO and N_2_ mixtures (Figure 1A). In the absence of CO, *C. necator* reached stationary phase after 24 h but with 15% (v/v) CO, the lag phase was increased, with cultures taking 30 h to reach stationary phase (Figure 1A) and displaying a statistically significant reduction in growth rate (*p* < 0.05; Table 1). This inhibitory effect was seen to an even greater extent in the presence of 30% and 50% (v/v) CO, with *C. necator* taking 42 h and 48 h, respectively, to reach stationary phase and both conditions significantly reducing the growth rate (*p* < 0.01 for both) (Table 1). For growth on CO_2_ + H_2_, F-MM grown pre-cultures were used to inoculate serum bottles containing minimal medium (MM) without organic carbon source and headspaces comprised of syngas-like mixtures with CO concentrations ranging from 0% to 50% (v/v) (Figure 1B). A significant reduction in growth rate and lag phase was observed when 15% CO was present in the headspace as compared to 0% CO (*p* = 0.00001) (Table 1), with cells taking 16 days longer to reach stationary phase (Figure 1B). When 30% and 50% (v/v) CO was present, *C. necator* H16 was unable to grow over the course of the experiment. These results suggested that under heterotrophic conditions CO, whilst inhibiting growth in a concentration-dependent manner, was still tolerated to some degree at relatively high concentrations, whereas under autotrophic conditions, cells were far more sensitive and growth was severely affected even at lower CO concentrations.

**TABLE 1:** Growth rates for *C. necator* H16 with increasing CO concentrations.

Figure/Experiment	Growth rate h^−1^
Figure 1A—heterotrophic growth	
0% CO	0.276 ± 0.035
15% CO	0.211 ± 0.033
30% CO	0.151 ± 0.027
50% CO	0.118 ± 0.021
Figure 1B—autotrophic growth	
0% CO	0.227 ± 0.008
15% CO	0.115 ± 0.055
30% CO	0.000 ± 0.000
50% CO	0.000 ± 0.000

To establish whether *C. necator* can evolve to tolerate high concentrations of CO adaptive laboratory evolution was carried out under both heterotrophic and autotrophic conditions as described below.

### Adaptive laboratory evolution to increase CO tolerance under heterotrophic conditions

Since growth was still observed for heterotrophic H16 cultures in the presence of 50% (v/v) headspace CO (Figure 1), this concentration was chosen for the conducted ALE experiments. An initial ALE experiment was carried out whereby a single H16 wild type colony was grown in F-MM, and then used to inoculate a serum bottle containing F-MM and a headspace of 50% CO/50% air (v/v), a growth condition hereafter referred to as 50% CO. This culture was grown to an OD_600_ of approximately 3, then reinoculated into a fresh serum bottle diluted to an OD_600_ of 0.01 every 3 days. The purpose of this pilot experiment was to establish whether CO tolerance of the H16 strain could be improved in principle and also the degree of growth improvement that might be expected within a reasonable timeframe. After eighty-one subcultures equivalent to an estimated 665 generations and carried out over 8 months, three isolates designated EA, EB, and EC were obtained from the evolved culture, and shown to display a clear growth advantage over the H16 parent strain under the employed CO growth regime, with a significantly higher growth rate and shorter lag phase but similar final OD (*p* > 0.05 for the ODs of all isolates) (Supplementary Figure S1).

**FIGURE 2:**
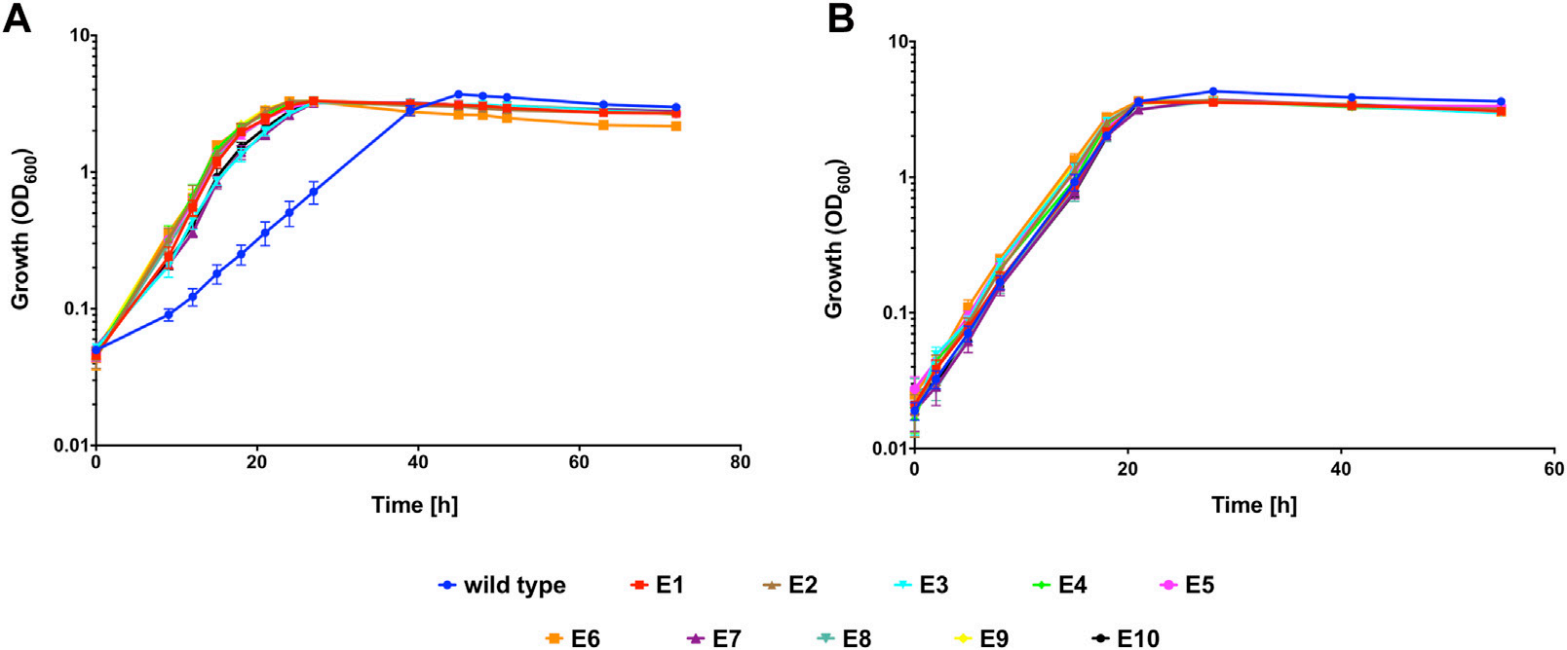
Heterotrophic growth of evolved *C. necator* isolates E1 to E10 compared to wild type *C. necator* H16 in the presence of CO. Cultures were grown at 30°C in 20 mL F-MM contained in 150 mL serum bottles under a 2 bar atm of **(A)** 50% CO/50% air (v/v) or **(B)** 50% N_2_/50% air (v/v). Blue circles, wild type; red squares, E1; brown triangles, E2; cyan inverted triangles, E3; green diamonds, E4; pink circles, E5; orange squares, E6; purple triangles, E7; turquoise inverted triangles, E8; yellow diamonds, E9; black circles, E10. Error bars represent the standard deviation of the mean for two independent replicates.

Having demonstrated that *C. necator* H16 could be evolved to a faster growing more CO tolerant phenotype, the experiment was repeated on a larger scale, with ten independently evolving lineages. For this, a single colony of *C. necator* H16 was cultured in F-MM. The genomic DNA of this culture was extracted and the remaining part used to inoculate ten separate pre-cultures in F-MM which were then independently subcultured in the presence of CO as described above, resulting in ten separate cultural lineages designated Evo1 to Evo10. This continued until a significant visible change in OD appeared within 18 h of re-inoculation, which would signify that the cells had adapted to the presence of CO. This was observed around the 15th round of re-inoculation, at which point stocks were generated for all cultures and preserved at −80°C. The evolved cultures were plated out and single colonies were isolated to represent defined strains designated isolate E1 to E10, according to their culture origin (Evo1 to Evo10, respectively). A growth comparison between the unevolved H16 parent strain and E1 to 10 was carried out under the same subculturing conditions in the presence and absence of 50% CO (Figure 2). Under the CO atmosphere, the evolved strains reached peak OD_600_ after 27 h and the wild type controls after 48 h (Figure 2A). There was a statistically significant difference between the growth rates of the evolved strains and the H16 wild type replicates (*p* < 0.01 for all strains; Table 2). By contrast, when grown under an N_2_ control atmosphere (consisting of 50% N_2_ and 50 air (v/v), hereafter referred to as 50% N_2_) both H16 parent strain and E1 to E10 isolates reached peak OD_600_ after 28 h (Figure 2B), with the latter not displaying a faster maximum growth rate to the former (*p* > 0.05 for all strains; Table 2). This indicated that isolated E1 to E10 had indeed adapted to the CO atmosphere rather than growth under serum flask conditions.

**TABLE 2:** Heterotrophic growth rates for evolved *C. necator* isolates E1 to E10 and wild type H16.

Figure/Experiment	Growth rate CO h^−1^	Growth rate N_2_ h^−1^
Figure 2		
E1	0.249 ± 0.024	0.234 ± 0.016
E2	0.250 ± 0.030	0.241 ± 0.031
E3	0.254 ± 0.022	0.230 ± 0.021
E4	0.258 ± 0.027	0.226 ± 0.016
E5	0.259 ± 0.016	0.235 ± 0.032
E6	0.272 ± 0.026	0.225 ± 0.024
E7	0.256 ± 0.028	0.239 ± 0.028
E8	0.263 ± 0.020	0.217 ± 0.023
E9	0.242 ± 0.023	0.254 ± 0.012
E10	0.254 ± 0.036	0.236 ± 0.017
wild type	0.118 ± 0.024	0.231 ± 0.015

The growth rate of the 8-month pilot isolates EA to EC was not significantly different to any of the obtained growth rates for E1 to E10 (*p* > 0.5 for all strains), therefore it was assumed that further ALE of these strains would not lead to further improvements in CO tolerance.

### Whole genome sequencing analysis of heterotrophically evolved CO resistant isolates

Illumina next-generation sequencing (NGS) was performed as described in the Materials & Methods for the evolved isolates E1 to E10, as well as isolates EA, EB and EC from the 8-month pilot evolution experiment (Supplementary Figure S1). For this, chromosomal DNA was isolated from each evolved isolate as well as the initial, unevolved parent strain (as present in the respective single colony-derived precultures) to be certain that identified differences in the former, when compared to the published H16 genome, were not already present in the latter. Following NGS, analysis of the obtained genome sequences was carried out using Qiagen CLC Genomics Workbench as described in the Methods, identifying several unique mutations that with high confidence were only present in the evolved strains E1 to E10 and EA to EC (Table 3).

**TABLE 3:** Comparison of mutations across *C. necator* E1—10 and *C. necator* A, B & C.

Locus tag^a^	Mutation^b^	Amino acid change	E1	E2	E3	E4	E5	E6	E7	E8	E9	E10	EA	EB	EC	Position^c^	Gene/encoded protein
H16_B1461	G → A	_	Yes	Yes	Yes	Yes	Yes	Yes	Yes	Yes	Yes	Yes	Yes	Yes	Yes	1638520	Intergenic, upstream of cytochrome *bd* ubiquinol oxidase operon
H16_A3118	Deletion: CACCGCGAGCGC	His-Arg-Glu-Arg deleted	—	Yes	Yes	Yes	Yes	—	—	—	Yes	—	—	Yes	Yes	3376140-3376151	Hybrid sensor histidine kinase/response regulator PhcR
H16_B0085	C → G	Ala→Gly	—	—	—	—	—	—	—	—	—	—	Yes	Yes	Yes	98474	Iron transporter FeoA
H16_A2071	Insertion: C	Frame shift	—	—	—	—	—	—	—	—	—	—	Yes	Yes	Yes	2253191	Hypothetical protein
H16_A3140	Deletion: G	_	—	—	—	—	—	—	—	Yes	Yes	—	—	—	—	3399139	Intergenic, upstream of H16_A3140
	Insertion: T	_	—	—	—	—	Yes	—	—	—	—	—	—	—	—	3399143	
H16_A3140	C → G	_	—	—	—	—	—	—	—	—	—	—	Yes	Yes	—	3400456	Intergenic, downstream of H16_A3140
	A → G	_	—	—	—	—	—	—	—	Yes	—	Yes	—	—	—	3400457	
	G → A	_	—	—	—	—	—	—	—	Yes	—	Yes	—	—	—	3400460	
H16_A3450	T → C	Ser→Pro	—	—	—	—	—	—	—	—	—	—	Yes	—	Yes	3730714	SB-like family cytochrome c maturation protein
H16_A3144	T → G	Leu→Arg	—	—	—	—	—	—	—	Yes	—	—	—	—	—	3403057	LysR family transcriptional regulator PhcA
	T → G	_	—	—	—	—		Yes		—	—	—	—	—	—	3403900	Intergenic region upstream of *phcA* gene
H16_A0387	A → C	Gln→Pro	Yes	—	—	—	—	—	—	—	—	—	—	—	—	405744	RNA polymerase sigma-54 factor
	C → A	Ser→STOP	—	—	—	—	—	Yes	Yes	—	—	—	—	—	—	404574	
H16_PHG126	G → A	Glu→Lys	—	—	—	—	—	—	—	—	—	—	—	Yes	—	125022	TonB-dependent receptor
	C → G	Asn→Lys	—	—	—	—	—	—	—	—	—	—	—	Yes	—	125377	
H16_B0573	A → G	Lys→Arg	—	—	Yes	—	—	—	—	—	—	Yes	—	—	—	645528	Membrane Protein
H16_A0146	Deletion: G	Frame shift	—	—	—	—	—	—	Yes	—	—	—	—	—	—	159883	Conserved hypothetical protein
H16_pHG019	A → T	Ile→Phe	—	—	—	Yes	—	—	—	—	—	—	—	—	—	16093	NtrC family transcriptional regulator
H16_A0195	T → G	His→Gln	—	—	—	—	—	—	—	—	—	—	—	Yes	—	208932	Putative GTPase (G3E family)
H16_A1404	C → A	_	—	—	—	—	—	—	—	—	—	—	—	Yes	—	1522049	Intergenic, upstream of H16_A1404
H16_A1439	G → A	Gly→Ser	—	—	—	—	—	—	—	—	—	—	—	Yes	—	1560730	Acetoacetyl-CoA reductase PhaB1

^a^ H16_A = chromosome 1, H16_B = chromosome 2, and PHG, megaplasmid pHG1.

^b^ Base changes always refer to the respective coding strand of the indicated locus, including when located up or downstream.

^c^ Position in the genome refers to the sequence given under Genbank accession numbers AM260479.1, AM260480.1 and AY305378.1 respectively.

Interestingly, a single point mutation (G→A) upstream of the cytochrome *bd* ubiquinol oxidase operon (hereafter referred to as cytochrome *bd*) was detected in all ten isolates E1 to E10 and in all three isolates EA to EC from the initial pilot culture (Table 3). A 12 bp deletion in the gene for hybrid sensor histidine kinase/response regulator PhcR was found in five of the E isolates (E2, E3, E4, E5 and E9) and two isolates (EB and EC) from the initial evolving culture (Table 3). The deletion removed the coding sequence for the peptide sequence His-Arg-Glu-Arg from this gene. A point mutation (T→G) was found upstream of the transcriptional activator PhcA encoding gene in one isolate (E6). A second point mutation within this region was found within the *phcA* gene itself (T→G) for isolate E8, changing a leucine to an arginine codon. Two point mutations were found within the RNA polymerase sigma factor 54 (*rpoN*) gene. A C→A transversion converted a serine codon into a stop codon and was found in two of the isolates (E6 and E7) and a A→C transversion changed a glutamine codon into a proline codon and was only seen on one occasion (E1) (Table 3).

The six mutations described above appeared to be the most likely candidates to contribute to CO resistance. Cytochrome *bd* ubiquinol oxidase II (encoded by *cydA2B2*) is one of two cytochrome *bd* terminal oxidases (the other encoded by *cydA1B1,* H16_B1177) of the *C. necator* electron transport chain (Pohlmann et al., 2006; Cramm, 2009), which may be resistant to CO. The mutation upstream of *cydA2B2* (G→A) may alter expression of the *cydA2B2* gene. To test this hypothesis, BPROM (Solovyev and Salamov, 2011) was used to predict bacterial sigma-70 promoter sequences. Indeed, this predicted the generation of a promoter sequence for this particular single base change. PhcA and PhcR are the transcriptional activator and regulator of the Phc quorum sensing system, where PhcR regulates activity of PhcA (Garg et al., 2000). It seemed reasonable to assume that genes controlled by this system may contribute to the lower CO tolerance seen for H16 parent strain. Similarly, genes under the control of RNA polymerase sigma-54 factor may also negatively affect CO tolerance. These six mutations were therefore chosen for further characterisation in the *C. necator* H16 background. Before embarking on a detailed analysis of the indicated loci and their potential involvement in CO resistance, it was however necessary to confirm the existence of the proposed mutations by independent means. This was achieved through amplification of the respective gene regions by PCR and subsequent Sanger sequencing which confirmed their presence in all cases. The other mutations found in some of the obtained isolates (Table 3) were deemed unlikely to be contributing significantly towards carbon monoxide resistance due to their location and observed frequency.

### Replication of the evolved heterotrophic CO resistant phenotype through targeted mutation of the wild type strain

To test whether the six chosen mutations increase CO tolerance in *C. necator*, a series of knock-out/knock-in plasmids derived from pLO3 (Lenz and Friedrich, 1998) were constructed to artificially recreate the individual mutations in the H16 wild type genome (plasmids pCWS01-10) (Supplementary Table S2). To achieve this, the following strategy was employed. First, a deletion of approximately 200 bp was introduced into the H16 genome at the position of the respective mutation. This deletion was then repaired with a sequence containing the given mutation but otherwise identical to the wild type. This generated knock-in mutant strains each containing the desired mutation and knock-out strains which could be further characterised if desired. The mutated regions for these strains were amplified by PCR and the resulting fragments purified and subjected to Sanger sequencing. This way, successful introduction of the chosen mutations could be confirmed. The generated strains were designated *C. necator cydA2B2* SNP, *C. necator phcR* 12 bp, *C. necator phcA* US, *C. necator rpoN* 5744 and *C. necator rpoN* 4574 for mutations in or upstream of *cydA2B2, phcR, phcA* and *RNA* polymerase sigma 54 factor, respectively. The knock-out stage of this method was unsuccessful for the mutation within the *phcA* gene and was therefore not further pursued.

**FIGURE 3:**
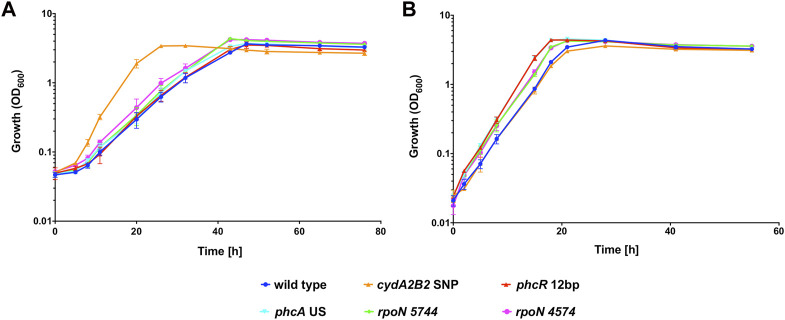
Heterotrophic growth of *C. necator* H16 and its single knock-in mutant derivatives in the presence of CO. Cultures were grown at 30°C in 20 mL F-MM contained in 150 mL serum bottles under a 2 bar atm of **(A)** 50% CO/50% air (v/v) or **(B)** 50% N_2_/50% air (v/v). Blue circles, wild type; orange triangles, *C. necator cydA2B2* SNP; red triangles, *C. necator phcR* 12 bp; cyan triangles, *C. necator phcA* US; green diamonds, *C. necator rpoN* 5744; pink circles, *C. necator rpoN* 4574. Error bars represent the standard deviation of the mean for three independent replicates.

Heterotrophic growth of the single knock-in mutants was compared with the H16 wild type in the presence and absence of 50% CO (Figure 3). Under the CO atmosphere, the wild type reached its peak OD_600_ after 48 h (Figure 3A). In contrast *C. necator cydA2B2* SNP reached stationary phase after 26 h and was the fastest growing mutant, displaying a significantly higher growth rate than the wild type (*p* = 0.0003; Table 4). *C. necator phcR* 12 bp and *C. necator phcA* US reached their peak OD_600_ at the same time as the wild type but *C. necator rpoN* 5744 and *rpoN* 4574 both appeared to display a slight growth advantage due to a slightly shorter lag phase, reaching peak OD_600_ at 43 h; however, maximum growth rates for all three aforementioned strains were not significantly different to that of the wild type (*p* > 0.05). The slightly improved growth of these mutants as seen in Figure 3A was due to achieving a slightly higher final OD_600_ and/or somewhat faster growth compared to the wild type in the later stages of growth. Under the N_2_ atmosphere, the wild type reached peak OD_600_ after 28 h (Figure 3B). Interestingly *C. necator cydA2B2* SNP reached its highest OD_600_ at the same time as the wild type and exhibited a similar growth rate, indicating there was no growth advantage for this strain in the absence of CO. In contrast, the remaining four strains appeared to grow slightly faster compared to the wild type. *C. necator phcR* 12 bp and *C. necator phcA* US reached peak OD_600_ after 19 h, and *C. necator rpoN* 5744 and *rpoN* 4574 after 22 h; however the maximum growth rate of these strains was not significantly higher than that of the wild type (*p* ≥ 0.1 for all). Similar to what was observed in the presence of CO, the improved growth of these mutants as seen in Figure 3B was due to somewhat faster growth in the later stages of the growth cycle. The results suggested that the analysed mutations other than the SNP found upstream of *cydA2B2* had no direct effect on CO resistance but generally improved *in vitro* growth performance under serum bottle conditions.

**TABLE 4:** Heterotrophic growth rates of the wild type H16 and its knock-in mutant derivatives.

Figure/Experiment	Growth rate CO h^−1^	Growth rate N_2_ h^−1^
Figures 3A, B		
wild type	0.123 ± 0.019	0.242 ± 0.014
*cydA2B2* SNP	0.274 ± 0.042	0.212 ± 0.036
*phcR* 12 bp	0.122 ± 0.017	0.249 ± 0.022
*phcA* US	0.138 ± 0.018	0.278 ± 0.018
*rpoN* 5744	0.136 ± 0.021	0.245 ± 0.016
*rpoN* 4574	0.139 ± 0.022	0.261 ± 0.047

### Characterisation of *cydA2B2*

The single point mutation (G→A) 210 bp upstream of the cytochrome *bd* operon *cydA2B2* appeared to be the only mutation that gave *C. necator* significantly increased tolerance to CO. Since the mutation was not in a protein-encoding region, it may be altering operon expression; therefore, further investigation into this operon was carried out, using three approaches. First, the upstream intergenic region of the operon was tested for promoter activity using an RFP-based reporter plasmid to elucidate whether introduction of the point mutation increased the level of expression as previously hypothesised. Second, the operon was overexpressed in an otherwise wild type background, again to determine the resulting effect on CO tolerance. Additionally, the two subunit-encoding genes of the operon were knocked out to determine how this impacted on CO tolerance.

To ascertain whether the G→A mutation influenced gene expression of *cydA2B2*, two RFP-based reporter plasmids were constructed containing the entire 616 bp upstream intergenic region of *cydA2B*2 and the backbone of pEH006 (Hanko et al., 2017). One plasmid harboured the G→A mutation in this region (pCWS13) whilst the other contained the wild type sequence (pCWS12), both replacing the arabinose-inducible pBAD promoter of pEH006. *C. necator* H16 derivatives each carrying one of the above plasmids were grown in F-MM alongside *C. necator* containing a positive control plasmid expressing the *rfp* gene in response to arabinose induction (pEH006) and an equivalent negative control vector lacking the pBAD promoter (pEH006E) (Hanko et al., 2017). The latter was used to correct for background fluorescence. These cultures were diluted back to an OD_600_ of 0.05 in fresh serum bottles containing F-MM and either 50% CO or 50% N_2_ in the headspace. *C. necator* pEH006 was supplemented with different arabinose concentrations (0 mM, 0.1 mM, 0.2 mM), so that the strength of the predicted promoter on pCWS13 could be compared to the output given by different inducer concentrations.

**FIGURE 4:**
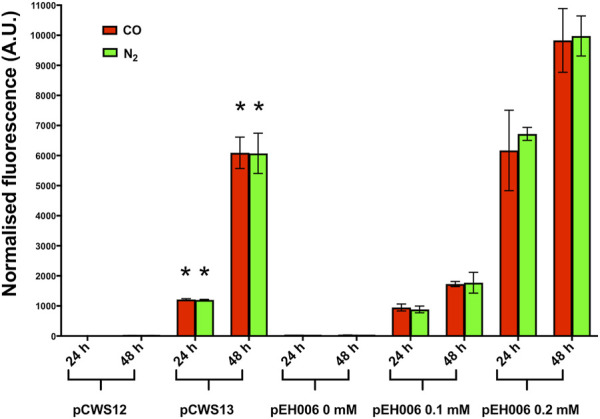
RFP expression of C. necator H16 containing either pCWS12, pCWS13 or pEH006. Cultures were grown at 30°C in 20 mL F-MM contained in 150 mL serum bottles under a 2 bar atm of 50% CO/50% air (v/v) (red bars) or 50% N_2_/50% air (v/v) (green bars). Expression of the *rfp* gene was monitored after 24 h and 48 h by determining RFP fluorescence. pCWS12 contained the wild type upstream region of *cydA2B2* linked to *rfp*; pCWS13 contained the mutated upstream region of *cydA2B2* linked to *rfp* and RFP expression of pEH006 was induced with the indicated arabinose concentrations of 0 mM, 0.1 mM and 0.2 mM. Average normalised fluorescence is given for each time point. Error bars represent standard deviations from the mean for three biological replicates. There was no significant difference between samples taken from CO or N_2_ grown cultures for *C. necator* pCWS12 (24 h, *p* = 0.98; 48 h, *p* = 0.64) and *C. necator* pCWS13 (24 h, *p* = 0.56; 48 h, *p* = 0.97). There was a significant difference (indicated by an asterisk) between *C. necator* pCWS13 and *C. necator* pCWS12 in the presence of CO and compared to the N_2_ control (*p* < 0.00001 for all time points).

Samples were taken at 24 and 48 h to measure fluorescence and optical density for each sample using a Tecan plate reader (Figure 4). After 24 h, in the presence of either CO or N_2_, *C. necator* pCWS13 produced approximately 1000-fold more fluorescence than *C. necator* pCWS12 and following 48 h around 500-fold more (*p* < 0.00001 for each time point) (Figure 4). Likewise, *C. necator* pCWS13 produced similar levels of fluorescence under both atmospheres. These results confirmed that the G→A mutation increased *cydA2B2* operon expression in *C. necator* regardless of whether CO was present or absent, possibly by increasing the promoter strength or by enhancing or inhibiting transcription factor binding in this intergenic region.

**FIGURE 5:**
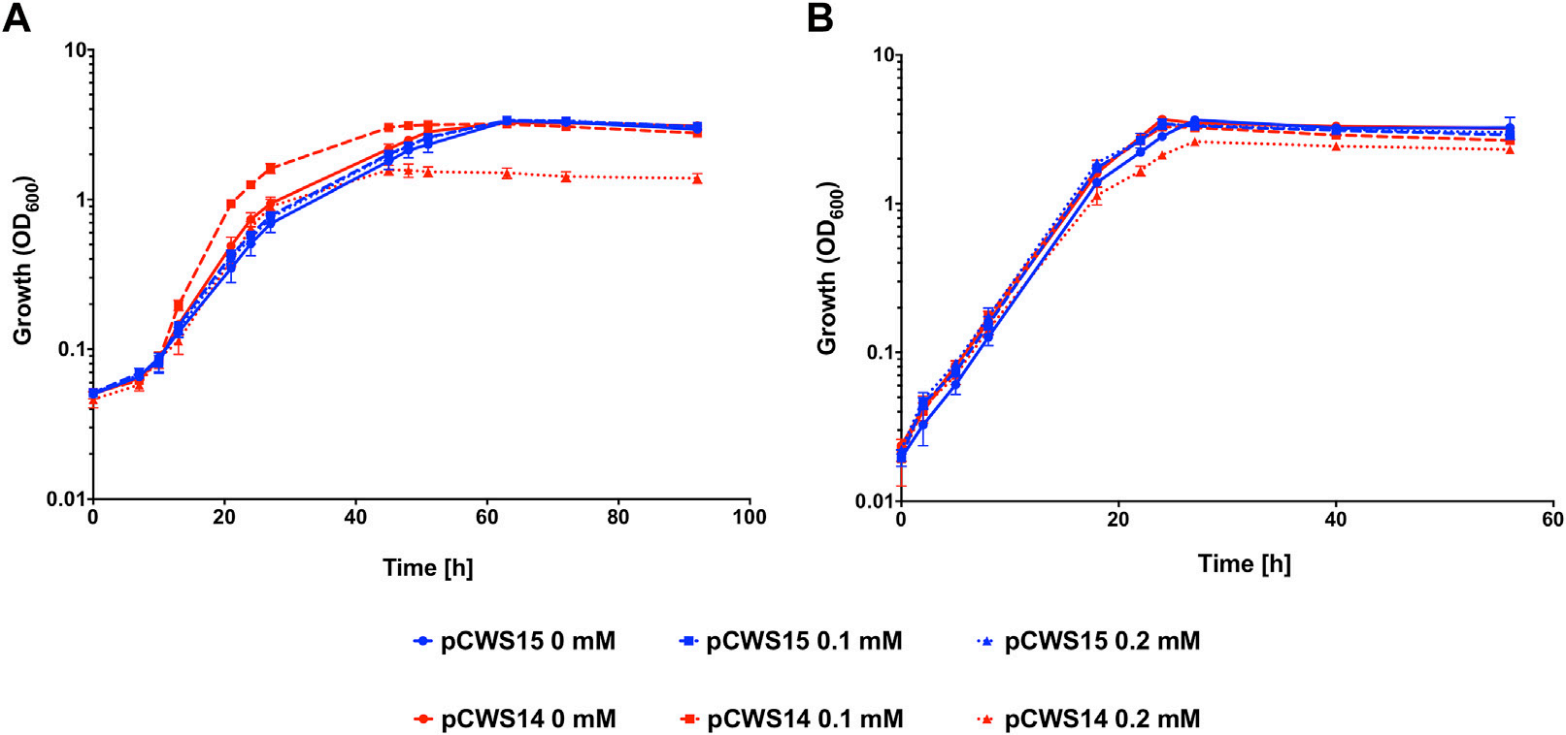
Heterotrophic growth of *C. necator* pCWS14 and *C. necator* pCWS15 in the presence of CO. Cultures were grown at 30°C in 20 mL F-MM contained in 150 mL serum bottles under a 2 bar atm of **(A)** 50% CO/50% air (v/v) or **(B)** 50% N_2_/50% air (v/v). Expression of *cydA2B2* from pCWS14 using the pBAD promoter was induced with the indicated arabinose concentrations: 0 mM, red line; 0.1 mM, red dashed line; 0.2 mM, red dotted line. pCWS15, which contained the arabinose-inducible pBAD promoter but lacked the *rfp* gene, was induced with the same arabinose concentrations: 0 mM, blue line; 0.1 mM, blue dashed line; 0.2 mM, blue dotted line. Error bars represent the standard deviation of the mean for three independent replicates.

To determine whether expression of *cydA2B2* would positively influence CO tolerance, an expression plasmid containing these genes was constructed and introduced into *C. necator*. This plasmid, pCWS14, was comparable to the arabinose inducible RFP reporter pEH006, but contained the *cydA2B2* operon in place of *rfp*. A derivative of pEH006, lacking the RFP gene (pCWS15) was also created and used as a control plasmid. The level of fluorescence produced by pCWS13 after 24 h was comparable to pEH006 induced with 0.1 mM and after 48 h to pEH006 induced with 0.2 mM arabinose (Figure 4); therefore, these concentrations were used to induce pCWS14, with the aim of achieving suitable levels of *cydA2B2* expression. In the presence of CO and without arabinose induction, *C. necator* pCWS14 reached peak OD_600_ after 51 h and *C. necator* pCWS15 after 63 h (Figure 5A). Under these conditions, *C. necator* pCWS14 did not display a significantly faster growth rate than the control (*p* = 0.09) (Table 5). Addition of 0.1 mM arabinose slightly improved growth of the *C. necator* pCWS15 control, but this was not statistically significant when compared to the growth rate obtained in the absence of arabinose (*p* > 0.05) (Figure 5A). However, the same concentration significantly increased the growth rate of *C. necator* pCWS14 (*p* = 0.006), which reached stationary phase after 45 h, 18 h earlier than *C. necator* pCWS15, presumably due to increased expression of *cydA2B2*. In the presence of 0.2 mM arabinose, *C. necator* pCWS15 displayed no further improvement in growth; however, *C. necator* pCWS14 exhibited reduced growth, reaching a lower peak OD_600_ than with 0 or 0.1 mM arabinose and exhibiting a reduced growth rate (Table 5). Taken together the obtained results suggested that induced expression of the cytochrome *bd* genes with 0.1 mM arabinose increased CO tolerance, whereas a further increase in expression in the presence of 0.2 mM arabinose had detrimental effects.

**TABLE 5:** Heterotrophic growth rates for wild type H16, H16 pCWS14, H16 pCWS15, *cydA2B2* KO, *cydA2B2* SNP and *cydA2B2* KI.

Figure/Experiment	Growth rate CO h^−1^	Growth rate N_2_ h^−1^
Figures 5A, B		
H16 pCWS14 0 mM arabinose	0.149 ± 0.015	0.211 ± 0.043
H16 pCWS14 0.1 mM arabinose	0.227 ± 0.023	0.226 ± 0.014
H16 pCWS14 0.2 mM arabinose	0.160 ± 0.016	0.216 ± 0.021
H16 pCWS15 0 mM arabinose	0.124 ± 0.022	0.230 ± 0.030
H16 pCWS15 0.1 mM arabinose	0.139 ± 0.019	0.252 ± 0.019
H16 pCWS15 0.2 mM arabinose	0.141 ± 0.010	0.232 ± 0.036
Figures 6A, B		
*cydA2B2* KO	0.094 ± 0.012	0.274 ± 0.029
wild type	0.123 ± 0.017	0.268 ± 0.011
*cydA2B2* SNP	0.270 ± 0.038	0.273 ± 0.026
*cydA2B2* KI	0.127 ± 0.016	0.272 ± 0.023

When grown under 50% N_2_, both *C. necator* pCWS15 and pCWS14 displayed no significant difference in growth rate with 0 mM, 0.1 mM or 0.2 mM arabinose (*p* > 0.05 for all conditions), all reaching stationary phase after 26 h (Figure 5B). However, in the presence of 0.2 mM arabinose *C. necator* pCWS14 displayed a reduction in peak optical density, although to a lesser extent than in the presence of CO (Figures 5A, B). A reduced peak optical density under 50% N_2_ was also observed when the *cydA1B1* operon was overexpressed in *C. necator* via pCWS17 (Supplementary Figure S2), encoding a second native cytochrome *bd* ubiquinol oxidase. However, cultures overexpressing *cydA1B1* at arabinose concentrations of 0.1, 0.2 and 0.3 mM in the presence of CO did not display an increased growth rate in comparison to the empty vector controls (*p* > 0.05 for all conditions).

**FIGURE 6:**
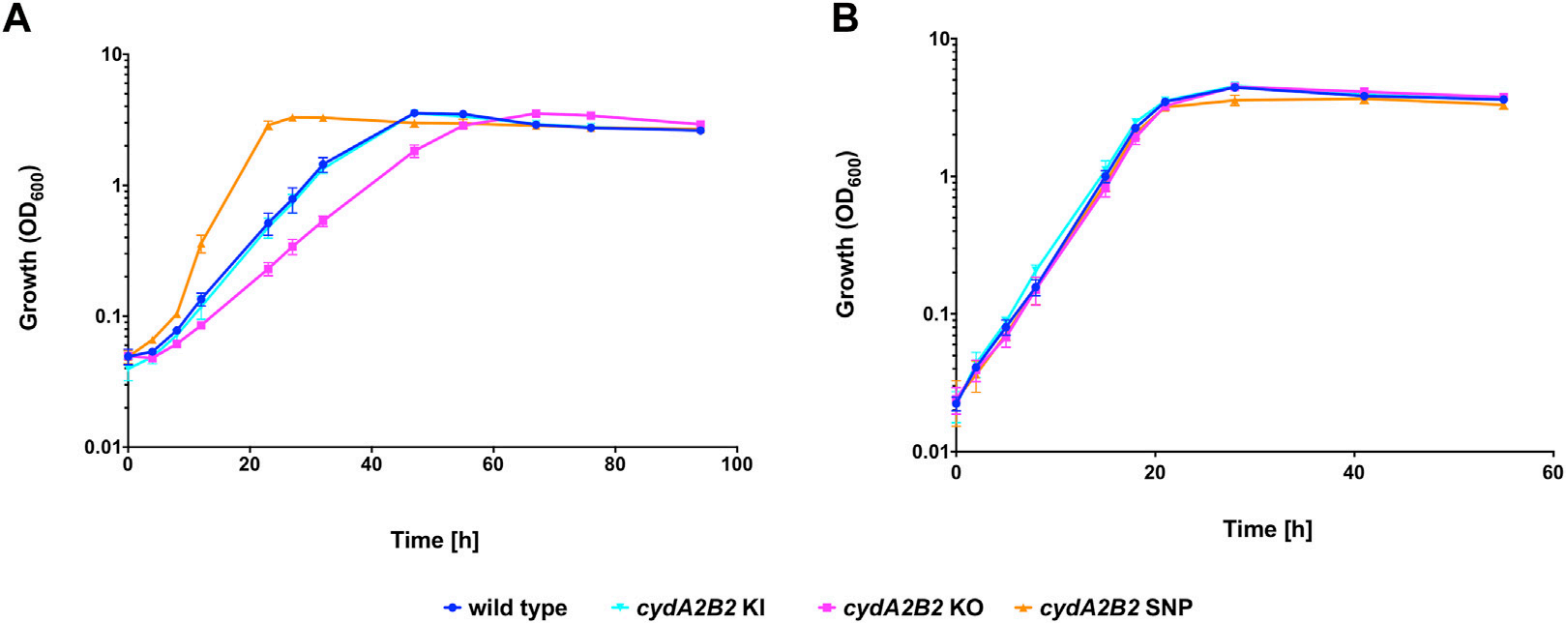
Heterotrophic growth in the presence of CO of *cydA2B2* KO, *cydA2B2* SNP, and *cydA2B2* KI strains compared to the H16 wild type. Cultures were grown at 30°C in 20 mL F-MM contained in 150 mL serum bottles under a 2 bar atm of **(A)** 50% CO/50% air (v/v) or **(B)** 50% N_2_/50% air (v/v). Blue circles, wild type; orange triangles, *cydA2B2* SNP; pink squares, *cydA2B2* KO; cyan inverted triangles, *cydA2B2* KI. Error bars represent the standard deviation of the mean for three independent replicates.

To investigate whether the lack of cytochrome *bd* impacts on the capacity of *C. necator* to tolerate CO, a knock-out plasmid derived from pLO3 was constructed (pCWS11) to delete the *cydA2B2* operon as described in the Methods. The obtained knock-out mutant was also genetically complemented using the knock-in plasmid pCWS16 to reintegrate *cydA2B2* into the original deletion site. Growth of the generated *cydA2B2* knock-out mutant (*C. necator cydA2B2* KO) was compared to that of the H16 wild type, complemented mutant (*C. necator cydA2B2* KI) and the generated, defined SNP knock-in strain *cydA2B2* SNP. Under a 50% CO atmosphere the wild type strain as well as *cydA2B2* KI reached peak OD_600_ after 47 h and the *cydA2B2* SNP strain after 27 h (Figure 6A). In contrast, the *cydA2B2* KO strain reached peak OD_600_ after 67 h and displayed an increased lag phase in comparison to the wild type strain. This indicated a somewhat reduced but not abolished CO tolerance resulting from *cydA2B2* inactivation. This was reflected by the strains’ obtained growth rates (Table 5), which was lower for the *cydA2B2* KO strain and increased for *cydA2B2* SNP as seen before (*p* = 0.027 and *p* = 0.002, respectively). The complemented mutant strain *cydA2B2* KI did not show a significant difference in growth rate to the wild type H16 strain (*p* = 0.469). Under a 50% N_2_ atmosphere, the wild type, *cydA2B2* SNP, *cydA2B2* KO and *cydA2B2* KI strains reached their peak OD_600_ after 28 h (Figure 6B). There was no significant difference between the growth rates of the mutant strains and the wild type strain under these conditions (*p* > 0.05); Table 5), indicating that a lack of cytochrome *bd* did not noticeably impact on the growth of *C. necator* in the absence of CO.

To investigate whether *cydA2B2* plays a role in CO resistance when *C. necator* is grown autotrophically, a growth comparison between the H16 wild type, *cydA2B2* SNP and E1 strains was performed with a syngas-like mixture containing 15% CO/65% H_2_/10% CO_2_/10% air (v/v). Neither *cydA2B2* SNP nor E1 displayed a significantly increased growth rate compared to the wild type (*p* > 0.05 for both strains) (Supplementary Table S4) (Supplementary Figure S3). These results suggest that the CO resistance conferred by cytochrome *bd* is limited to heterotrophic growth.

### Adaptive laboratory evolution to increase CO tolerance under autotrophic conditions

To observe whether *C. necator* H16 can adapt to autotrophic growth in the presence of high CO concentrations, a single colony was grown in an F-MM pre-culture, then diluted to an OD_600_ of 0.01 in a serum bottle containing MM and a headspace of 15% CO/65% H_2_/10% CO_2_/10% air (v/v), based on the established tolerance threshold shown in Figure 1B.

**FIGURE 7:**
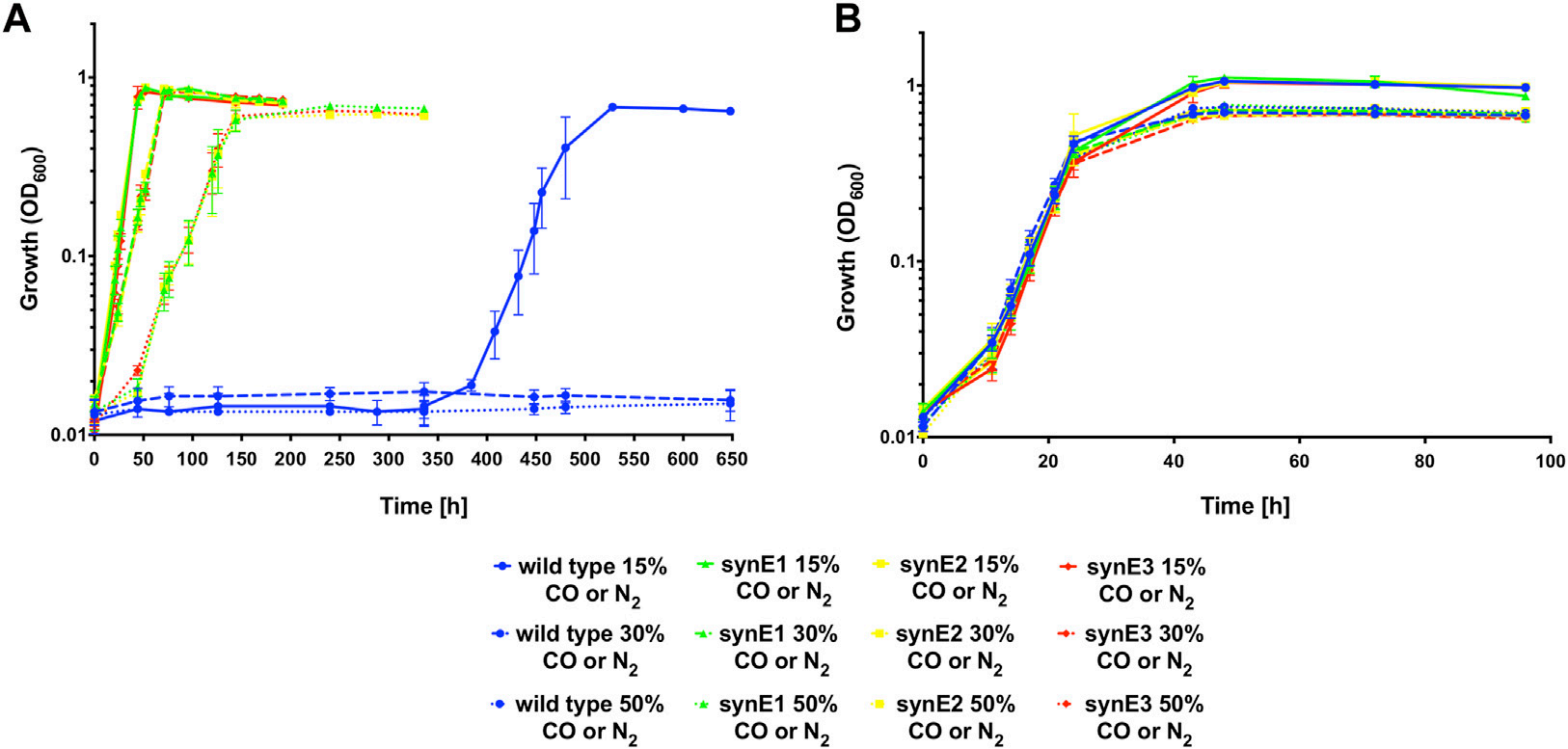
Autotrophic growth of *C. necator* synE isolates compared to the H16 wild type under syngas-like atmospheres with increasing CO concentrations. Cultures were grown at 30°C in 25 mL MM contained in 250 mL serum bottles under a 2.4 bar atm of **(A)** 15% CO/65% H_2_/10% CO_2_/10% air (v/v) (full lines), or 30% CO/50% H_2_/10% CO_2_/10% air (v/v) (dashed lines), or 50% CO/30% H_2_/10% CO_2_/10% air (v/v) (dotted lines), and **(B)** 15% N_2_/65% H_2_/10% CO_2_/10% air (v/v) (full lines), or 30% N_2_/50% H_2_/10% CO_2_/10% air (v/v) (dashed lines), or 50% N_2_/30% H_2_/10% CO_2_/10% air (v/v) (dotted lines). Blue circles, wild type; green triangles, synE1; yellow squares, synE2; red circles, synE3. Error bars represent the standard deviation of the mean for two independent replicates.

These cultures were grown to an OD_600_ of approximately 0.7 over 18 days, then reinoculated to an OD_600_ of 0.01 into a fresh serum bottle containing higher CO headspace concentrations of either 30% and 50%, respectively (represented by gas mixtures consisting of 30% CO/50% H_2_/10% CO_2_/10% air (v/v) and 50% CO/30% H_2_/10% CO_2_/10% air (v/v), respectively). These cultures were now able to reach stationary phase after 4 days and therefore continued subculturing with 50% CO was carried out as described above until a shift occurred following the eighth round of reinoculation, whereby the culture took 3 days to reach stationary phase. When compared to the H16 parent strain as previously described, following plating and testing of single colonies, the selected evolved isolates, designated synE1, synE2 and synE3, displayed a significantly increased growth rate (*p* < 0.0001 for each isolate) when grown in the presence of 15% CO (Figure 7A). The evolved isolates were also able to grow with 30% and 50% CO, in contrast to the wild type (Figure 7A). In the control experiment, where the different CO concentrations were replaced with N_2_, there was no significant difference between the wild type and the evolved isolates (*p* > 0.05) (Table 6; Figure 7B).

**TABLE 6:** Autotrophic growth rates for wild type H16, synE1, synE2, synE3, *hoxH* SNP and *hoxH* KO strains.

Figure/Experiment	Growth rate CO h^−1^	Growth rate N_2_ h^−1^
Figures 7A, B		
wild type 15% CO or N_2_	0.048 ± 0.015	0.205 ± 0.029
synE1 15% CO or N_2_	0.107 ± 0.024	0.223 ± 0.019
synE2 15% CO or N_2_	0.111 ± 0.015	0.210 ± 0.039
synE3 15% CO or N_2_	0.104 ± 0.009	0.219 ± 0.027
wild type 30% CO or N_2_	0.000 ± 0.000	0.223 ± 0.027
synE1 30% CO or N_2_	0.065 ± 0.010	0.252 ± 0.019
synE2 30% CO or N_2_	0.067 ± 0.014	0.204 ± 0.018
synE3 30% CO or N2	0.067 ± 0.017	0.252 ± 0.019
wild type 50% CO or N_2_	0.000 ± 0.000	0.211 ± 0.023
synE1 50% CO or N_2_	0.037 ± 0.019	0.223 ± 0.035
synE2 50% CO or N_2_	0.038 ± 0.018	0.225 ± 0.025
synE3 50% CO or N_2_	0.039 ± 0.012	0.228 ± 0.032
Figures 8A, B		
wild type	0.041 ± 0.006	0.231 ± 0.014
*hoxH* SNP	0.086 ± 0.016	0.229 ± 0.030
*hoxH* KO	0.000 ± 0.000	0.047 ± 0.008
synE1	0.103 ± 0.005	0.236 ± 0.016

It was also desirable to obtain a strain that exhibited increased CO tolerance under both autotrophic and heterotrophic conditions, and hence the *cydA2B2* SNP strain was subjected to the same autotrophic evolution regime described above. The autotrophic growth of three isolates from the evolved culture, designated *cydA2B2* Evo1, *cydA2B2* Evo2 and *cydA2B2* Evo3, was compared against the synE1, *cydA2B2* SNP and wild type H16 strains on MM under a syngas-like atmosphere containing 15% CO as described above (Supplementary Figure S4A). The *cydA2B2* Evo isolates and synE1 reached peak OD_600_ after 52 h and both the wild type and *cydA2B2* SNP strains after 20 days. There was a significant difference in growth rate between the *cydA2B2* Evo isolates and *cydA2B2* (*p* < 0.01 for each isolate) as well as the wild type (*p* < 0.01 for each isolate), indicating clear adaptation to the syngas atmosphere (Supplementary Table S4). There was no significant difference between *cydA2B2* Evo and synE1 (*p* = 0.781).

Additionally, to confirm whether the synE1 strain had also developed heterotrophic CO resistance, the growth of this strain on F-MM and 50% CO/50% air (v/v) was compared to the wild type, *cydA2B2* SNP and *cydA2B2* Evo isolates (Supplementary Figure S4B). SynE1 displayed no significant difference in growth rate to the wild type (*p* = 0.445), reaching stationary phase at the same time (Supplementary Figure S4). Whereas, both *cydA2B2* SNP and the *cydA2B2* Evo isolates grew significantly faster than the wild type and synE1 (*p* < 0.01 each comparison).

### Whole genome sequencing analysis following autotrophic growth

Illumina NGS with read mapping and variant calling was performed as described in the Materials & Methods for the syngas-evolved isolates synE1, synE2 and synE3 to identify potential mutations that may have caused their evolved CO tolerant phenotype. Indeed, all of these isolates had acquired two unique mutations not present in the H16 parent strain. A C→T transition was found within the reading frame of the gene encoding the beta subunit of the NAD-reducing hydrogenase *hoxH*, one of the two large subunits of the soluble hydrogenases in *C. necator* (Jugder et al., 2015). The other mutation was a G→A transition in the translation initiation factor gene *infA* (Table 7). The former mutation converts a proline to serine in amino acid position 288, and the latter produces a stop codon in place of tryptophan. These mutations were separately confirmed by Sanger sequencing following PCR amplification of the respective regions. The mutation in the *hoxH* codon region was thought likely to contribute to increased CO resistance when *C. necator* is grown with syngas, even though the hydrogenases of *C. necator* have previously been described as CO tolerant and to contain CO as a ligand in their active site (Buhrke et al., 2005; Burgdorf et al., 2005; Bürstel et al., 2016). Furthermore, following PCR amplification and Sanger sequencing of their *hoxH* gene regions, isolates *cydA2B2* Evo1, *cydA2B2* Evo2 and *cydA2B2* Evo3 were also shown to contain this particular serine generating mutation. By contrast, in addition to NGS, Sanger sequencing of the respective PCR-amplified gene regions from synE1, synE2 and synE3 independently confirmed that these isolates did not contain the *cydA2B2* upstream mutation. This suggests that *cydA2B2* expression does not further increase CO tolerance when combined with the *hoxH* mutation, a finding also supported by the very similar growth of the *cydA2B2* Evo and the synE isolates (Supplementary Figure S4A).

**TABLE 7:** Mutations found in isolates synE1, synE2 and synE3.

Locus tag^a^	Mutation	Amino acid change	synE1	synE2	synE3	Position	Encoded protein
PHG091	C → T	Proline to serine (Pro288Ser)	Yes	Yes	Yes	83726	HoxH, subunit of the NAD-reducing soluble hydrogenase
H16_A3118	G → A	Tryptophan to STOP	Yes	Yes	Yes	2647724	Translation initiation factor IF-1

^a^ Position in the genome refers to the sequence given under Genbank accession numbers AM260479.1 and AY305378.1, respectively.

### Replication of the evolved autotrophic CO resistant phenotype

**FIGURE 8:**
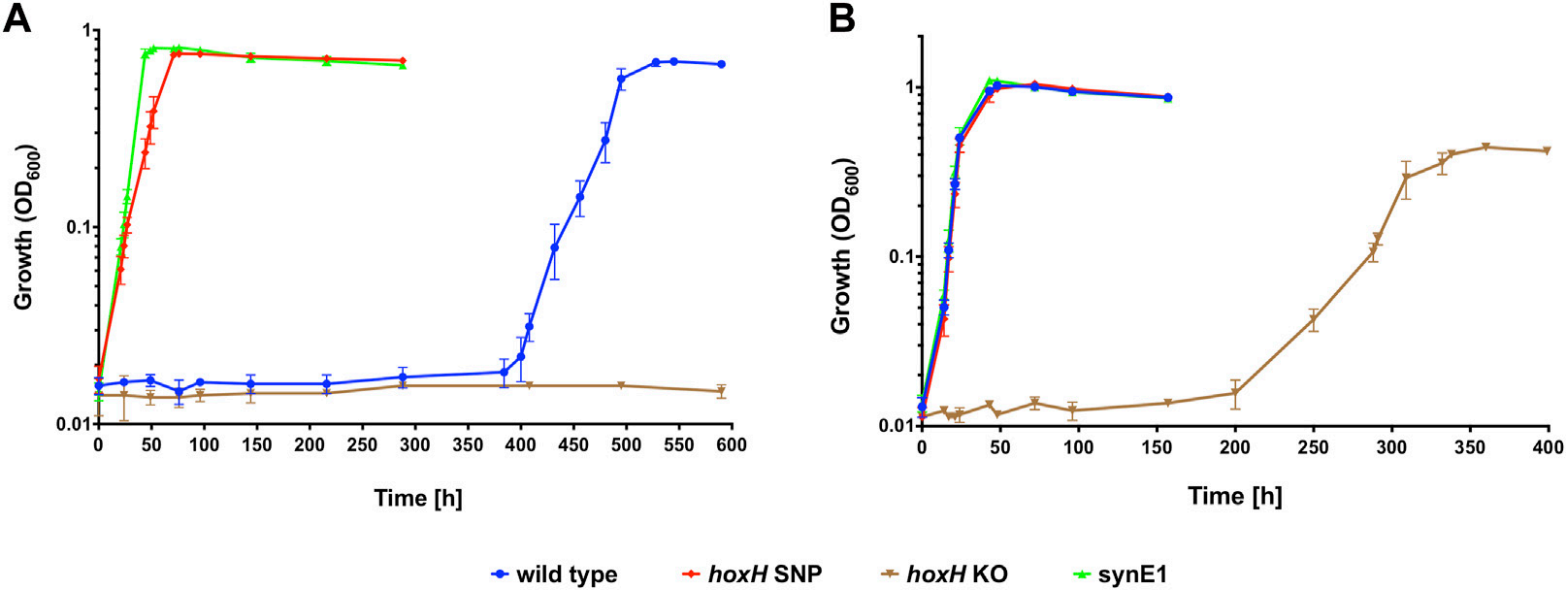
Autotrophic (A) and heterotrophic growth (B) of *C. necator hoxH* SNP, *hoxH* KO and synE1 strains compared to the H16 wild type under a syngas-like atmosphere. Cultures were grown at 30°C in 25 mL MM contained in 250 mL serum bottles under a 2.4 bar atm of **(A)** 15% CO/65% H_2_/10% CO_2_/10% air (v/v) or **(B)** 15% CO/65% H_2_/10% CO_2_/10% air (v/v). Blue circles, wild type; red squares, *hoxH* SNP; inverted brown triangles, *hoxH* KO; green triangles, synE1. Error bars represent the standard deviation of the mean for three independent replicates.

The identified *hoxH* point mutation was engineered into the H16 wild type strain as described previously using the knock-out /knock-in method with plasmids derived from pLO3 (Supplementary Table S2). This generated the strains *hoxH* KO and *hoxH* SNP. Despite numerous attempts, *infA* could not be modified in this manner and therefore the SNP found in this gene could not be introduced into the wild type strain or *hoxH* SNP. The protein encoded by this gene, IF-1, is a highly conserved translational protein (Cummings and Hershey, 1994), and most likely essential to H16. Autotrophic growth of the generated *hox* mutant strains was compared with the H16 wild type and synE1 in the presence and absence of CO (Figure 8A). All strains were grown with a syngas mixture containing either 15% CO or 15% N_2_ (v/v) to an OD_600_ of 0.01. In the presence of CO, *hoxH* SNP displayed a significantly faster growth rate than the wild type and was able to reach peak OD_600_ after 72 h (Figure 8A). Furthermore, the growth rate of this strain was not significantly different to synE1 (*p* = 0.30). In contrast, as might be expected due to its soluble hydrogenase deficiency, *hoxH* KO was not able to grow within the time frame of the experiment. In the absence of CO, only *hoxH* KO displayed a significantly different growth rate to the wild type (*p* = 0.00001) (Figure 8B), taking 15 days to reach stationary phase. The enhanced growth of *hoxH* SNP suggested that the point mutation in this strain improved tolerance to CO, as in the *C. necator* synE isolates, presumably through the change of amino acid from proline to serine in the HoxH subunit.

## Discussion

A key issue when considering syngas and similar industrial waste gases as feedstocks for industrial fermentations is their high content of CO and associated toxicity. Bacteria show varying degrees of sensitivity to CO, with respiration and growth being inhibited in the presence of the gas (Cypionka and Meyer, 1982; King, 2003; Desmard et al., 2009; Nobre et al., 2009), although generally much less so than mammals. The mechanism underlying high CO tolerance as observed for some bacteria are insufficiently understood and only few attempts have been made to increase the resilience of industrially relative strains through ALE, with a focus on strictly anaerobic Gram-positive bacteria (Shen et al., 1999; Kang et al., 2020; Jin et al., 2022). The aim of this study was to isolate and characterise mutant derivatives of *C. necator* H16 that have adapted to growth in the presence of high CO concentrations, to gain insights into potential molecular and physiological resistance mechanisms as well as strategies to engineer better performing strains.

Wild type *C. necator* H16 was already remarkably tolerant to the gas under the tested heterotrophic conditions, being able to grow with 50% CO in the headspace albeit with extended lag phases and reduced growth rates, in agreement with previous reports (Cypionka and Meyer, 1982). Under autotrophic conditions, however, CO-mediated growth inhibition was more severe, representing a bottleneck for syngas-based applications with possible impacts on yields, titres and productivity.

Heme-containing proteins are major targets for CO binding and inhibition. Studies with aerobic bacteria have shown that CO decreased their respiratory rates due to binding to the terminal oxidases in their respiratory chains (Cypionka and Meyer, 1982; Robb and Techtmann, 2018). There is also evidence that the effects of the gas are global (Wareham et al., 2016), with CO affecting various non-heme targets such as proteins that contain non-heme iron or other transition metal centres, including hydrogenases (Wu and Wang, 2005; Lubitz et al., 2014). Finding that the evolved H16 strains carried mutations affecting soluble hydrogenase and expression of cytochrome *bd* was therefore not entirely unexpected. Importantly the observed effects were reproduced when the respective mutations were introduced into the unevolved H16 parent strain, confirming that they were indeed responsible for the observed phenotype.

Whilst not assessed in this study, one must assume that the cytochrome *bd* encoded by *cydA2B2* is intrinsically more resistant to CO poisoning than other terminal oxidases potentially expressed under the tested conditions, including a homologous oxidase encoded by *cydA1B1* whose plasmid-based expression did not improve CO tolerance. Whilst expression from the native *cydA2B2* promoter was insufficient to achieve the high CO tolerance observed for the evolved strains, deletion of the encoding operon had a detrimental effect on CO resistance, suggesting that the enzyme nevertheless contributed to wild type tolerance even though expression levels based on the employed *rfp-*reporter were very low. This may have been a result of the chosen sampling points. Cytochrome *bd* oxidase is known to have high affinity for oxygen and is generally employed under stressful conditions such as oxygen-limitation or iron deficiency (Ingledew and Poole, 1984; Poole and Cook, 2000; Trutko et al., 2003; Borisov et al., 2009; Borisov et al., 2013; Borisov et al., 2021) and *C. necator* has been proposed to produce *bd*-type cytochromes in in the late log phase (Cramm, 2009). Increased expression of *cydA2B2* did not, however, improve autotrophic CO-tolerance. The operon is not usually expressed under these conditions (Kohlmann et al., 2011) and presumably one or more of the other terminal oxidases present during autotrophic growth exhibited a sufficient degree of CO tolerance.

Overall, our findings are in line with a growing body of evidence showing that the enzyme contributes to CO tolerance in a range of phylogenetically diverse bacteria. Increased production of cytochrome *bd* in response to CO exposure was recently reported for *M. smegmatis* and appears to be a major contributor to the observed high tolerance of the organism, the mutation resulting in its inactivation resulting in an increased lag phase under CO conditions (Bayly et al., 2021). Similarly, the *E. coli cydAB* operon encoding cytochrome *bd-*I was upregulated in response CO-releasing molecules (CORMS) (Davidge, et al., 2009; Wareham et al., 2018), but also by the free gas (Wareham et al., 2016), consistent with a physiological study showing that *E. coli* engineered to produce cytochrome *bd*-I as the only terminal oxidase showed the highest resistance to CO-releasing CORM-3 (Jesse et al., 2013). How the reported higher *in vitro* sensitivity of *E. coli* cytochrome *bd-*I and cytochrome *bd-*II (as compared to cytochrome *bo’*) fits into this picture (Forte et al., 2019) has yet to be established.

Bacterial “standard” [FeFe] and [NiFe] hydrogenases are usually strongly inhibited by CO (Lubitz et al., 2014). A notable exception is a group of oxygen tolerant [Ni-Fe] hydrogenases found in *C. necator*, *E. coli* and *Aquifex aeolicus* which are not significantly affected in their activity by CO (Schneider et al., 1979; Vincent et al., 2005; Lukey et al., 2010; Pandelia et al., 2010). For *C. necator* H16 this includes both membrane-bound (MBH) and soluble hydrogenase (SH). Hence, it was intriguing to find that a single base change in *hoxH*, encoding the larger, NiFe active site carrying subunit of the heterodimeric hydrogenase moiety of SH, had such a marked effect on autotrophic CO tolerance. This mutation, which was present in both evolved lines (starting from the H16 parent and *cydA2B2* SNP strains, respectively), changed proline 288 to a serine and this change was independently confirmed to confer increased CO tolerance by engineering it into the H16 wild type. This suggests that at least under *in vivo* conditions SH tolerance to CO can be further improved. In future studies it would be interesting to establish how the proline to serine mutation impacts on HoxH’s structure and specifically its catalytic NiFe center.

During heterotrophic ALE, several loci including those encoding the putative quorum sensing system Phc and the alternative sigma factor RpoN (sigma 54) accumulated mutations in several of the independently evolved lines, suggesting that these changes conferred a fitness benefit under the employed conditions. Since the tested SNPs and deletions did not increase CO tolerance when introduced into the unevolved H16 parent strain, it seems reasonable to assume that they represented adaptations to the specific serum bottle conditions used for the ALE experiments.

Previous work by Heinrich et al. (2018) has shown that *C. necator* H16 can be metabolically engineered to utilise CO derived carbon by introducing and expressing a heterologous CO dehydrogenase. Employing a similar approach for our evolved strains, it would be interesting to test whether this improves growth and CO incorporation of syngas-fed cultures.

## Data Availability

The raw NGS datasets presented in this study can be found in the NCBI Sequence Read Archive (SRA; https://www.ncbi.nlm.nih.gov/sra) under the following accession numbers: SRX19746595; SRX19746596; SRX19746597; SRX19746598; SRX19746599; SRX19746600; SRX19746601; SRX19746602; SRX19746603; SRX19746604; SRX19746605; SRX19746606; SRX19746607; SRX19746608; SRX19746609; SRX19746610; SRX19749121.
