# Supplementary material for: Improving carbon monoxide tolerance of Cupriavidus necator H16 through adaptive laboratory evolution
Source: Front Bioeng Biotechnol. 2023 Apr 24;11:1178536. doi: 10.3389/fbioe.2023.1178536 (PMC10164946; doi:10.3389/fbioe.2023.1178536)
Supplement: Supplementary file 1 [file Table1.DOCX]

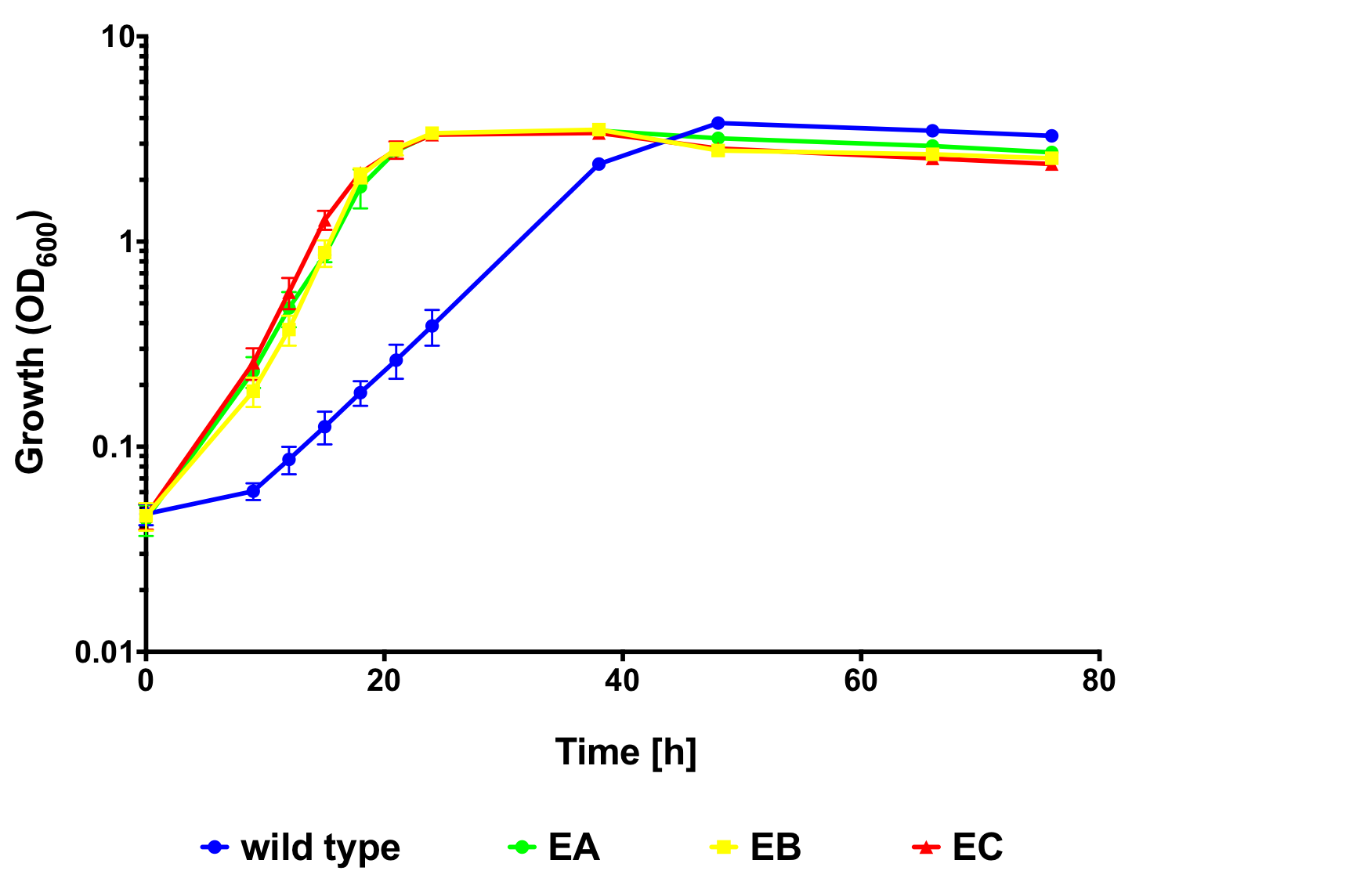


**Additional file 1: Figure S1. Heterotrophic growth of 8-month evolved *C. necator* H16 isolates compared to the wild type in the presence of CO.** Cultures were grown at 30°C in 20 ml F-MM contained in 150 ml serum bottles under a 2 bar atmosphere of 50% CO / 50% air (v/v). Wild type, blue circles; EA, green circles; EB, yellow squares; EC, red triangles. Error bars represent the standard deviation of the mean for two independent replicates.

**
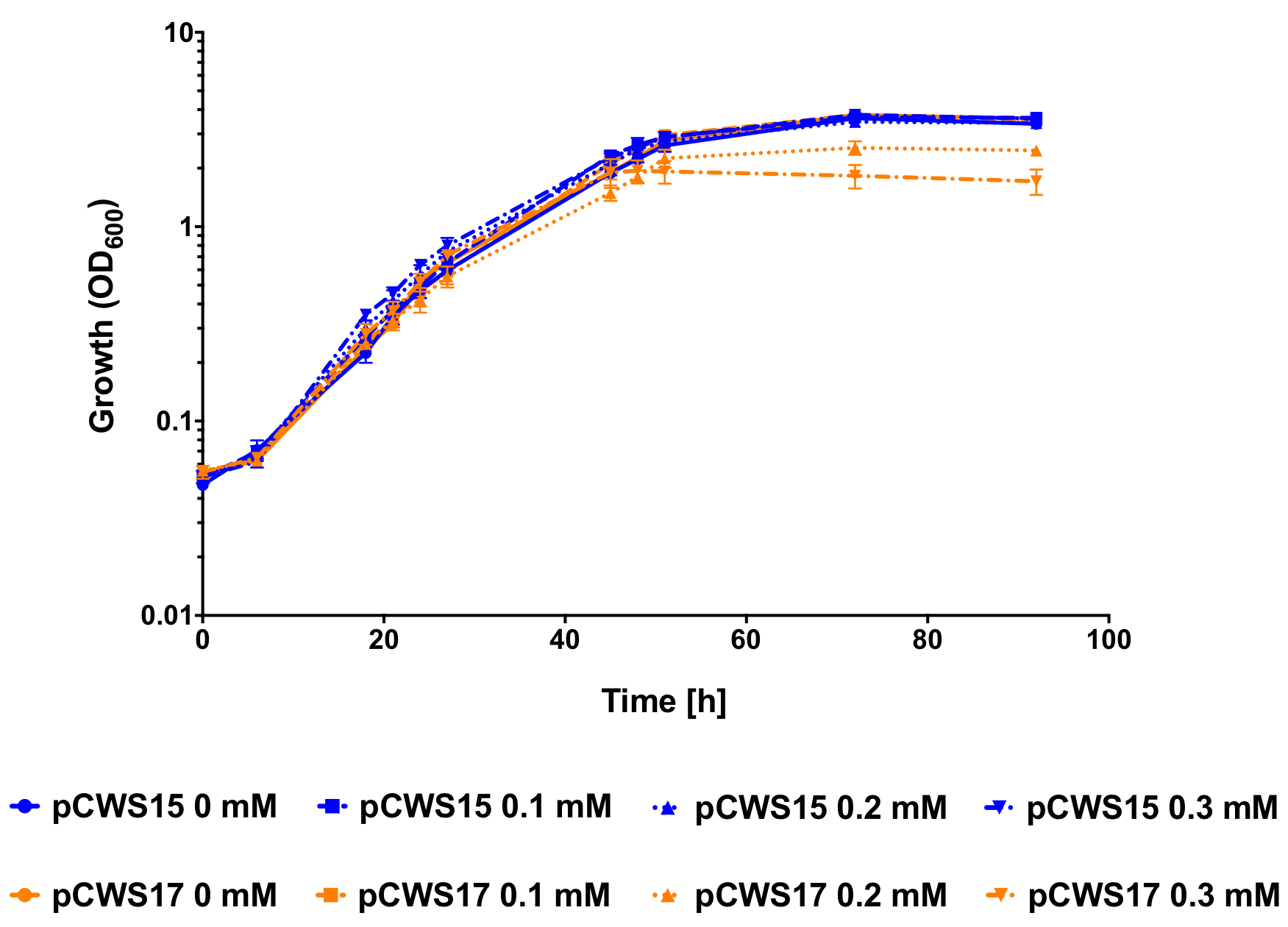
**

**Additional file 2: Figure S2. Heterotrophic growth of C. necator pCWS17 and C. necator pCWS15.** Cultures were grown at 30°C in 20 ml F-MM contained in 150 ml serum bottles under a 2 bar atmosphere of (**a**) 50% CO / 50% air (v/v) or (**b**) 50% N_2_ / 50% air (v/v). Expression of cydA1B1 from pCWS17 using the pBAD promoter was induced with the indicated arabinose concentrations: 0 mM, orange line; 0.1 mM, orange dashed line; 0.2 mM, orange dotted line; 0.3 mM, orange dotted and dashed line. pCWS15 which contained the arabinose-inducible pBAD promoter but lacked the rfp gene was induced with the same arabinose concentrations: 0 mM, blue line; 0.1 mM, blue dashed line; 0.2 mM, blue dotted line; 0.3 mM, blue dotted and dashed line. Error bars represent the standard deviation of the mean for three independent replicates.

**
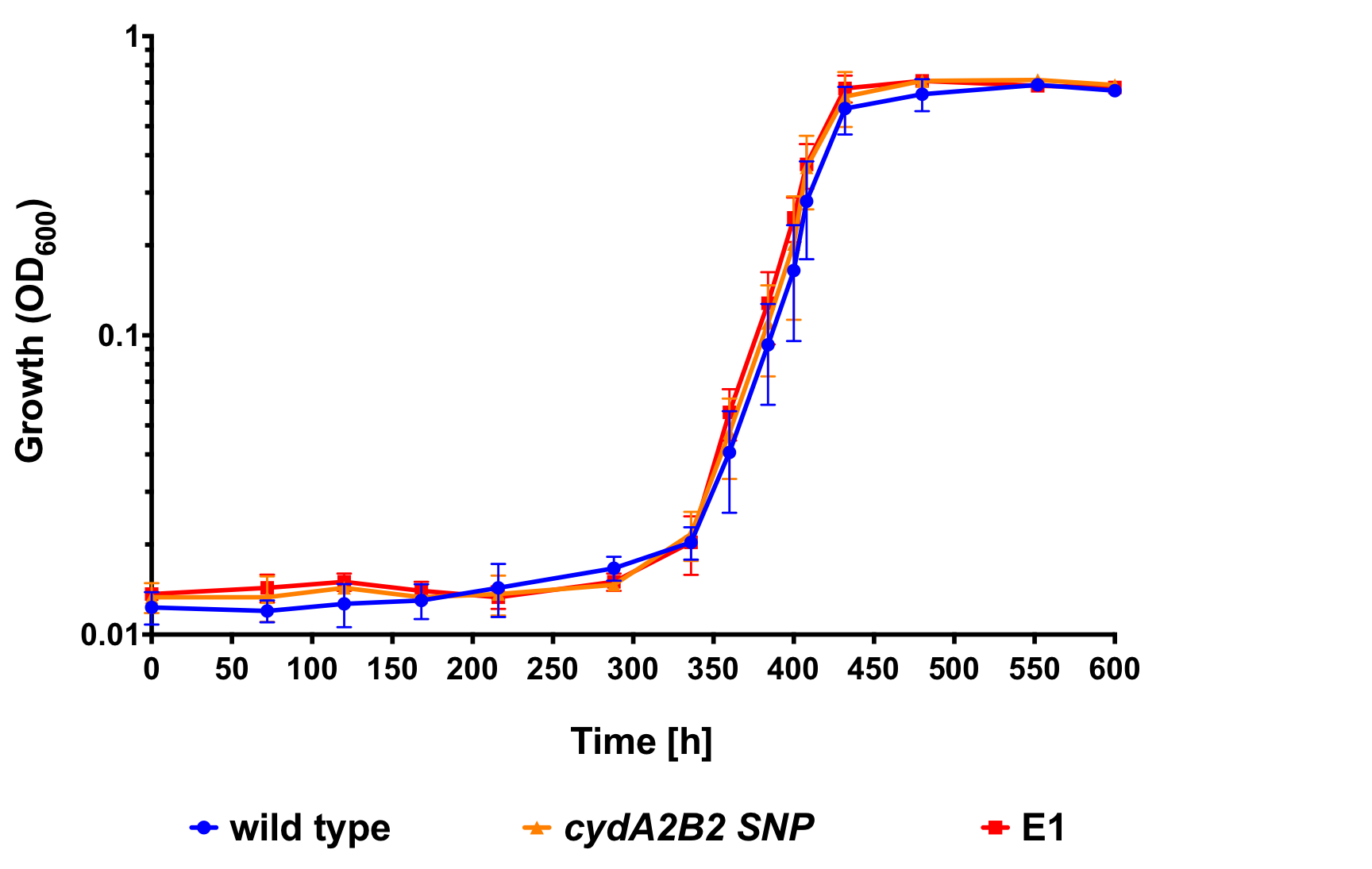
**

**Additional file 3: Figure S3. Autotrophic growth of H16 wild type, *cydA2B2* SNP and H16 E1 strains on syngas.** Cultures were grown at 30°C in 25 ml MM contained in 250 ml serum bottles under a 2.4 bar atmosphere of 15% CO, 65% H_2_, 10% CO_2_ and 10% air (v/v). Blue circles, wild type; red squares, *cydA2B2* SNP; purple triangles, E1. Error bars represent the standard deviation of the mean for three independent replicates.

**
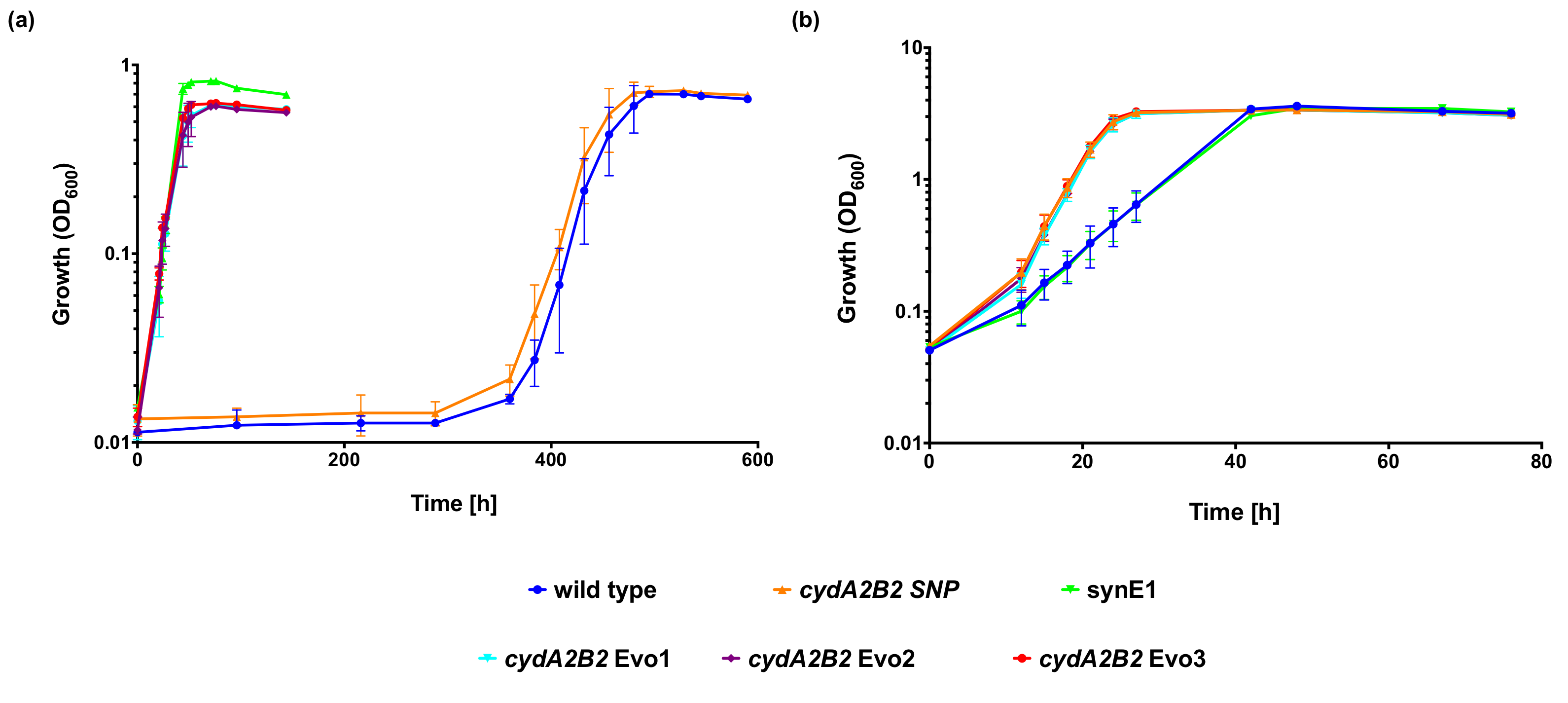
**

**Additional file 4: Figure S4: Autotrophic growth on syngas (a) and heterotrophic growth on fructose (b) in the presence of CO of H16 wild type, synE1, *cydA2B2* SNP and *cydA2B2* Evo isolates.** Cultures were grown at 30°C in (**a**) 25 ml MM contained in 250 ml serum bottles under a 2.4 bar atmosphere of 15% CO, 65% H_2_, 10% CO_2_ and 10% air (v/v) or (**b**) in 20 ml F-MM contained in 150 ml serum bottles under a 2 bar atmosphere of 50% CO / 50% air (v/v). Blue circles, wild type; inverted green triangles, synE1; orange triangles, *cydA2B2* SNP; cyan circles, *cydA2B2* Evo1; red circles, *cydA2B2* Evo2; purple triangles, *cydA2B2* Evo3. Error bars represent the standard deviation of the mean for three independent replicates.

**Additional file 5: Supplementary Table S1. Strains used and generated in this study**

| **Strain** | **Genotype / Description** | **Reference or source** |
| --- | --- | --- |
| H16 | *Cupriavidus necator* H16 wild type strain (DSM 428) | German Collection of Micro-organisms and Cell Cultures, Braunschweig, Germany (DSMZ) |
| EA | Isolated CO resistant H16 derivative from heterotrophic ALE; for genotype see Table 3 | This study |
| EB | Isolated CO resistant H16 derivative from heterotrophic ALE; for genotype see Table 3 | This study |
| EC | Isolated CO resistant H16 derivative from heterotrophic ALE; for genotype see Table 3 | This study |
| E1 - E10 | Isolated CO resistant H16 derivative from heterotrophic ALE; for genotype see Table 3 | This study |
| *cydA2B2* SNP KO | H16 derivative with deletion upstream of *cydA2B2* | This study |
| *cydA2B2* SNP | H16 derivative with SNP generated upstream of *cydA2B2* | This study |
| *phcR* | H16 derivative with *phcR* gene deleted | This study |
| *phcR* 12bp | H16 derivative with 12 bp (4 amino acid) deletion in *phcR* | This study |
| *phcA* KO | H16 derivative with upstream *phcA* region deleted | This study |
| *phcA* SNP | H16 derivative with SNP generated upstream of *phcA* | This study |
| *rpoN* 5744 KO | H16 derivative with part of the open reading frame of the RNA polymerase sigma-54 factor gene deleted | This study |
| *rpoN* 5744 | H16 derivative with SNP generated in the RNA polymerase sigma-54 gene (position 405744) | This study |
| *rpoN* 4574 KO | H16 derivative with part of the open reading frame of the RNA polymerase sigma-54 gene deleted | This study |
| *rpoN* 4574 | H16 derivative with SNP generated in the RNA polymerase sigma-54 gene (position 404574) | This study |
| *cydA2B2* KO | H16 derivative containing knock out of *cydA2B2* gene | This study |
| synE1 | Isolated CO resistant H16 derivative H16 derivative from autotrophic ALE, see Table 7 for genotype | This study |
| synE2 | Isolated CO resistant H16 derivative from autotrophic ALE, see Table 7 for genotype | This study |
| synE3 | Isolated CO resistant H16 derivative from autotrophic ALE, see Table 7 for genotype | This study |
| *cydA2B2* Evo1 | Isolated CO resistant *cydA2B2* SNP derivative from autotrophic ALE | This study |
| *cydA2B2* Evo2 | Isolated CO resistant *cydA2B2* SNP derivative from autotrophic ALE | This study |
| *cydA2B2* Evo3 | Isolated CO resistant *cydA2B2* SNP derivative from autotrophic ALE | This study |
| *hoxH* KO | H16 derivative containing knock out of *hoxH* | This study |
| *hoxH* SNP | H16 derivative with SNP generated in the HoxH gene (position 83726) | This study |
| *E. coli* DH5α | F^-^ φ80*lac*ZΔM15 Δ(*lac*ZYA-*arg*F)U169 *deo*R *recA1* *endA1 hsdR17*(r_k_^-^, m_k_^+^) *pho*A *sup*E44 *thi*-1 *gyr*A96 *relA1* λ- | (Grant *et al.* 1990) |
|  |  |  |
| *E. coli* S17-1 | *recA thi pro* *hsdR^-^ hsdM^+^* RP4::2-Tc::Mu::Km Tn7 *λpir*, Tp^R^ Sm^R^ | (Simon *et al.* 1983) |
|  |  |  |

Grant SG, Jessee J, Bloom FR, Hanahan D. Differential plasmid rescue from transgenic mouse DNAs into Escherichia coli methylation-restriction mutants. Proc Natl Acad Sci USA. 1990;87(12):4645-9.

Simon R, Priefer U, Pühler A. A broad host range mobilization system for invivo genetic engineering: transposon mutagenesis in Gram-negative bacteria. Bio/Technology. 1983;1:784-91.

**Additional file 6: Supplementary Table S2. Plasmids used and generated in this study**

| **Plasmid** | **Genotype / Description** | **Reference or source** |
| --- | --- | --- |
| pLO3 | Suicide vector used for deletion of genes in *C. necator*; carries *sacB* gene and Tet resistance cassette | (Lenz and Friedrich, 1998) |
| pCWS01 | Knock-out plasmid for upstream region of cytochrome *bd* ubiquinol oxidase *(cydA2B2)* | This study |
| pCWS02 | Knock-in plasmid to generate a defined SNP within the *cydA2B2* upstream region | This study |
| pCWS03 | Knock-in plasmid for inserting *phcR* with a defined 12 bp deletion into a larger *phcR* knock-out | This study |
| pCWS04 | Knock-out plasmid for in frame *phcR* knockout | This study |
| pCWS05 | Knock-out plasmid for the upstream region of *phcA* | This study |
| pCWS06 | Knock-in plasmid for the upstream region of *phcA* containing defined SNP | This study |
| pCWS07 | Knock-out plasmid for part of the open reading frame of the RNA polymerase sigma factor-54 gene (position 405744) | This study |
| pCWS08 | Knock-in plasmid to generate a defined SNP within the RNA polymerase sigma factor-54 gene containing novel SNP (position 405744) | This study |
| pCWS09 | Knock-out plasmid for an in-frame section of the RNA polymerase sigma factor-54 gene (position 404574) | This study |
| pCWS10 | Knock-in plasmid to generate a defined SNP within RNA polymerase sigma factor-54 gene containing novel SNP (position 404574) | This study |
| pCWS11 | Plasmid for deletion of *cydA2B2* | This study |
| pEH006 | RFP expressing plasmid with an arabinose-inducible system | (Hanko et al., 2017) |
| pEH006E | Derivative of pEH006 lacking the arabinose-inducible promoter | (Hanko et al*.,* 2017) |
| pCWS12 | Derivative of pEH006 containing upstream intergenic region of *cydA2B2* replacing the arabinose inducible pBAD promoter | This study |
| pCWS13 | Derivative of pEH006 containing upstream intergenic region of cytochrome *bd* with altered SNP replacing the arabinose inducible pBAD promoter | This study |
| pCWS14 | pEH006 with RFP gene replaced with *cydA2B2* downstream of the arabinose-inducible promoter | This study |
| pCWS15 | pEH006 lacking the RFP gene | This study |
| pCWS16 | Plasmid for integration of *cydA2B2* to genetically complement *cydA2B2* KO | This study |
| pCWS17 | pEH006 derivative with RFP gene replaced with *cydA1B1* downstream of the arabinose-inducible pBAD promoter | This study |
| pCWS18 | Plasmid for deletion of *hoxH* | This study |
| pCWS19 | Plasmid for integration of *hoxH* containing defined SNP | This study |
| pCWS20 | Plasmid for deletion of *infA* | This study |
| pCWS21 | Plasmid for deletion of *infA* containing defined SNP | This study |

Hanko EKR, Minton NP, Malys N. Characterisation of a 3-hydroxypropionic acid-inducible system from *Pseudomonas putida* for orthogonal gene expression control in *Escherichia coli* and *Cupriavidus necator*. Sci Rep. 2017;7(1):1724.

Lenz O, Friedrich B. A novel multicomponent regulatory system mediates H_2_ sensing in *Alcaligenes eutrophu*s. Proc Natl Acad Sci USA. 1998;95(21):12474-9.

**Additional file 7: Supplementary Table S3. Primers used in this study**

| **Oligonucleotide** | **Purpose in this study** | **Sequence** |
| --- | --- | --- |
| PHA_F | Confirmation of successful ligations into pLO3 | AGTGGAACGAAAACTCACGTTAAGGG |
| PHA_R | Confirmation of successful ligations into pLO3 | ACACGGTGCCTGACTGCG |
| HsdR_N F | To confirm *C. necator* H16 identity | ACAACCGCTTCACAGTGGTC |
|  |  |  |
| HsdR_N R | To confirm *C. necator* H16 identity | CTTGCGCTGATTGCTGTATC |
| CytoD_LHA F | To amplify left homology arm of *cydA2B2* upstream region for cloning into pLO3 | GATCCTTTTAATTCGAGCTCGGTACCCGGGAGCATCGGCAACATGACGG |
| CytoD_LHA R | To amplify left homology arm of *cydA2B2* upstream region for cloning into pLO3 | GAGGAGATTGCGCGGGATCACAGTC |
| CytoD_RHA F | To amplify right homology arm of *cydA2B2* upstream region for cloning into pLO3 | GACTGTGATCCCGCGCAATCTCCTCGCGCCTAGCCTGAC |
| CytoD_RHA R | To amplify right homology arm of *cydA2B2* upstream region for cloning into pLO3 | AATTAGCTTGCATGCCTGCAGGTCATGCAGCAGCTGCCAG |
| CytoD SNP F | To amplify right homology arm containing altered nucleotide in *cydA2B2* upstream region | ACGCTTGTCCATGGTATCCTGTCCCGCAATTGACCC |
| CytoD SNP R | To amplify left homology arm containing altered nucleotide in *cydA2B2* upstream region | GGACAGGATACCATGGACAAGCGTAAGGACT |
| CytoD SNP det F | Confirmation of single and double crossover of pCWS01 and pCWS02 | TGCAAGACGACCGTACTTTCGG |
| CytoD SNP det R | Confirmation of single and double crossover of pCWS01 and pCWS02 | GCGTGCTTCCAGGAACGC |
| CytoD seq F | Confirmation of single crossover of pCWS01 and pCWS02 | GGTGATGATCCAGGAAACCTTCAAGGG |
| CytoD seq R | Confirmation of single crossover of pCWS01 and pCWS02 | CGATCAGCACCATCCACAGCG |
| HisKin_LHA F | To amplify left homology arm of *phcR* 12bp deletion region for cloning into pLO3 | CACCTAGATCCTTTTAATTCGAGCTCCATTCGGTTCGGTCATCGGTCA |
| HisKin_LHA R | To amplify left homology arm of *phcR* 12bp deletion region for cloning into pLO3 | CACCGCGAGCGCACCCTGCGCCAGCACCGGCTGATGG |
| HisKin_RHA F | To amplify right homology arm of *phcR* 12bp deletion region for cloning into pLO3 | CGCAGGGTGCGCTCGCGGTGGCGCTCGGCGGCGCCTGCCAGCACCTCG |
| HisKin_RHA R | To amplify right homology arm of *phcR* 12bp deletion region for cloning into pLO3 | GCAGGTTTAAACAGTCGACTCTAGACCGACACCCTGGTGCTG |
| HisKin Del det F | Confirmation of single and double crossover of pCWS03 | CTCATGCGCCAGGAAGGC |
| HisKin Del det R | Confirmation of single and double crossover of pCWS03 | CTTCCGCATCCTGGAAAAGCC |
| HisKin seq SC F | Confirmation of single crossover of pCWS04 and pCWS04 | GCTGGAAATACTTGACTGCAGTCGC |
| HisKin seq SC R | Confirmation of single crossover of pCWS04 and pCWS04 | GCGCTCGATTCGTCGATCCG |
| PhcR Ko LHA F | To amplify left homology arm of *phcR* for cloning into pLO3 | TCCTTTTAATTCGAGCTCGGTACCCGGGGGCTGCACCAGGTACTCGG |
| PhcR Ko LHA R | To amplify left homology arm of *phcR* for cloning into pLO3 | AAGCAGGCACAGTCGAAAGCATAGGAGTGACCG |
| PhcR Ko RHA F | To amplify right homology arm of *phcR* for cloning into pLO3 | GCTTTCGACTGTGCCTGCTTCGCTTCGC |
| PhcR Ko RHA R | To amplify right homology arm of *phcR* for cloning into pLO3 | GCAGGTTTAAACAGTCGACTCTAGAGTCTACACGTTCATGAACGAGCG |
| PhcR seq F | Confirmation of single and double crossover of pCWS04 | GCACGGCACAGGATGGC |
| PhcR seq R | Confirmation of single and double crossover of pCWS04 | GCTGAACCTGGTCATCTTCGC |
| PhcA US SNP LHA F | To amplify left homology arm of *phcA* upstream region for cloning into pLO3 | GGATCTTCACCTAGATCCTTTTAATTCGAGCTCCGAGCATGGTGTTCAGTTCCG |
| PhcA US SNP LHA R | To amplify left homology arm of *phcA* upstream region for cloning into pLO3 | GGATGTACGAGTGCATGTTGCCGAAGCTTTTAGTGATCTTCACCG |
| PhcA US SNP RHA F | To amplify right homology arm of *phcA* upstream region for cloning into pLO3 | TCGGTGAAGATCACTAAAAGCTTCGGCAACATGCACTCGTACATCCG |
| PhcA US SNP RHA R | To amplify right homology arm of *phcA* upstream region for cloning into pLO3 | GCAGGTTTAAACAGTCGACTCTAGAGCTTCCGCAGTAAAGGAAAACGC |
| PhcA US SNP F | To amplify right homology arm of *phcA* upstream region with altered nucleotide for cloning into pLO3 | TTTCAACGGCGTCTTACTAGTGTAAGAGGAAAAAACAAGCTCGG |
| PhcA US SNP R | To amplify left homology arm of *phcA* upstream region with altered nucleotide for cloning into pLO3 | TTTTTTCCTCTTACACTAGTAAGACGCCGTTGAAATCTGATGTGC |
| PhcA US SNP det F | Confirmation of single and double crossover of pCWS05 and pCWS06 | GCGGATCGTCAAAGATTTCACGC |
| PhcA US SNP det R | Confirmation of single and double crossover of pCWS05 and pCWS06 | GCAGGCGGAAATGTTCAGGC |
| PhcA US seq F | Confirmation of single and double crossover of pCWS05 and pCWS06 | GGCAGTCCGACAAACTCGC |
| PhcA US seq R | Confirmation of single and double crossover of pCWS05 and pCWS06 | AGCTGGTACGACAACGAGTGGG |
| RNApol 5744 LHA F | To amplify left homology arm of *RNApol* 5744 region for cloning into pLO3 | GGATCTTCACCTAGATCCTTTTAATTCGAGCTCGCCTCGGCATCACATCCG |
| RNApol 5744 LHA R | To amplify left homology arm of *RNApol* 5744 region for cloning into pLO3 | ATGCAGCGGCCACTGACGGAAAACCCTCTGC |
| RNApol 5744 RHA F | To amplify right homology arm of *RNApol* 5744 region for cloning into pLO3 | TTTCCGTCAGTGGCCGCTGCATCACATGCG |
| RNApol 5744 RHA R | To amplify right homology arm of *RNApol* 5744 region for cloning into pLO3 | TGCAGGTTTAAACAGTCGACTCTAGAGAAGCCTTCCGTCGAAGCC |
| RNApol 5744 SNP F | To amplify right homology arm of *RNApol* 5744 region with altered nucleotide for cloning into pLO3 | TGGGAAAGGCGGAGCGGTAGCGACGGTTTCATGCG |
| RNApol 5744 SNP R | To amplify left homology arm of *RNApol* 5744 region with altered nucleotide for cloning into pLO3 | ATGAAACCGTCGCTACCGCTCCGCCTTTCCCAGCATCTGG |
| RNApol 5744 5 seqF | Confirmation of single and double crossover of pLO3_*RNApol 5744* KO and KI | GCCGGACTCACCCTTTGCG |
| RNApol 5744 5 seqR | Confirmation of single and double crossover of pLO3_*RNApol 5744* KO and KI | GCCAGAAACGCCTGAACGC |
| RNApol 4574 LHA F | To amplify left homology arm of *RNApol* 4574 region for cloning into pLO3 | GGATCTTCACCTAGATCCTTTTAATTCGAGCTCCCAGGATTCCCTCTCTTTGTATGGC |
| RNApol 4574 LHA R | To amplify left homology arm of *RNApol* 4574 region for cloning into pLO3 | CATCCAGCAGCCTTGATCAAGCAACTGATAG |
| RNApol 4574 RHA F | To amplify right homology arm of *RNApol* 4574 region for cloning into pLO3 | TTGATCAAGGCTGCTGGATGTTCTTGATCAG |
| RNApol 4574 RHA R | To amplify right homology arm of *RNApol* 4574 region for cloning into pLO3 | TGCAGGTTTAAACAGTCGACTCTAGAGCTGACGACGACAGCTATGGC |
| RNApol 4574 SNP F | To amplify right homology arm of *RNApol* 4574 region with altered nucleotide for cloning into pLO3 | GGGAGATGGTTTACTCGTGTAAACCCAGTGTATCGG |
| RNApol 4574 SNP R | To amplify left homology arm of *RNApol* 4574 region with altered nucleotide for cloning into pLO3 | ACTGGGTTTACACGAGTAAACCATCTCCCGGGTGACG |
| RNApol SNP det F | Confirmation of single and double crossover of pCWS07, pCWS08, pCWS09 and pCWS10 | GTCGGAAAGGGGATTCCTCGG |
| RNApol SNP det R | Confirmation of single and double crossover of pCWS07, pCWS08, pCWS09 and pCWS10 | GCCTACATCATCAGCGAAGGC |
| RNApol 4574 seqF | Confirmation of single and double crossover of pCWS07, pCWS08, pCWS09 and pCWS10 | AGATACATGGTGATGCAATGGTGCC |
| RNApol 4574 seqR | Confirmation of single and double crossover of pCWS07, pCWS08, pCWS09 and pCWS10 | CTCGGTCAACCTGCAGAGCG |
| CytoD inter US F | To amplify *cydA2B2* upstream intergenic region for cloning into pEH006 | ATATGACGTCGCGCCACCCCGGCTTCCG |
| CytoD inter US R | To amplify *cydA2B2* upstream intergenic region for cloning into pEH006 | ATATATCATATGCGATGTGCTCTCCGTGGGGTGGC |
| CytoD Exp F | To amplify *cydA2B2* for cloning into pEH006 | AATTCAAAAGATCTTTTAAGAAGGAGATATACAATGTACGGACTCACAGCC |
| CytoD Exp R | To amplify *cydA2B2* for cloning into pEH006 | TGGAGATCCTTACTCGAGTTTGTCAGTGGTACCCCTCCCC |
| Cbd op del LHA F | To amplify left homology arm for the in frame knock out of *cydA2B2* for cloning into pLO3 | GGATCTTCACCTAGATCCTTTTAATTCGAGCTGCACAAGCTGGCCATGGG |
| Cbd op del LHA R | To amplify left homology arm for the in frame knock out of *cydA2B2* for cloning into pLO3 | CCTGGCCATCAGTGGCGTACATCGATGTGCTCTCCG |
| Cbd op del RHA F | To amplify right homology arm for the in frame knock out of *cydA2B2* for cloning into pLO3 | AGAGCACATCGATGTACGCCACTGATGGCCAGGATCCC |
| Cbd op del RHA R | To amplify right homology arm for the in frame knock out of *cydA2B2* for cloning into pLO3 | GCAGGTTTAAACAGTCGACTCACGTCGGTGATCCAGCG |
| HoxH SNP F | To amplify right homology arm of *hoxH* region with altered nucleotide for cloning into pLO3 | GAGCTACATGAAGTTCTCCTACCTCAAGGAGC |
| HoxH SNP R | To amplify left homology arm of *hoxH* region with altered nucleotide for cloning into pLO3 | CTCCTTGAGGTAGGAGAACTTCATGTAGCTCC |
| HoxH det F | Confirmation of single and double crossover of pCWS19 | GACGACAAGCACATCGTCCG |
| HoxH det R | Confirmation of single and double crossover of pCWS19 | AGCAGTTCTTTGACCACCTCCG |
| HoxH seq F | Confirmation of single and double crossover of pCWS19 and pCWS18 | CGATCAATCGCTACGATTGATTAACGG |
| HoxH seq R | Confirmation of single and double crossover of pCWS19 and pCWS18 | AGCACTGAAAGCACCTTTGGC |
| HoxH seq F2 | Confirmation of single and double crossover of pCWS18 | GATCTTCAGCATCAGCCATCGG |
| hoxH seq R2 | Confirmation of single and double crossover of pCWS18 | TCAGCGGTTTGATGCTTTCCG |
| HoxH KO LHA F | To amplify left homology arm of *hoxH* upstream region for cloning into pLO3 | GGATCTTCACCTAGATCCTTTTAATTCGAGCTCAAGTCAAAGAGGCGGTTGCG |
| hoxH KO LHA R | To amplify left homology arm of *hoxH* upstream region for cloning into pLO3 | GCAAGGGTCACATAGGGAAACTCACATAACAAACCCTCCGTTAATCAATCG |
| HoxH KO RHA F | To amplify right homology arm of *hoxH* downstream region for cloning into pLO3 | GGTTTGTTATGTGAGTTTCCCTATGTGACCCTTGCC |
| HoxH KO RHA R | To amplify right homology arm of *hoxH* downstream region for cloning into pLO3 | TGCAGGTTTAAACAGTCGACTCTAGAAGCTCGCCAACCAGGTCCC |
| InfA KO LHA F | To amplify left homology arm of *infA* upstream region for cloning into pLO3 | GGATCTTCACCTAGATCCTTTTAATTCGAGCTCTTCACGGTGCCCCAGGC |
| InfA KO LHA R | To amplify left homology arm of *infA* upstream region for cloning into pLO3 | CCGTGCAGAAACTCACAATCTGTCTTCCTTTTCGGCAGCC |
| InfA KO RHA F | To amplify right homology arm of *infA* downstream region for cloning into pLO3 | CGAAAAGGAAGACAGATTGTGAGTTTCTGCACGGCCC |
| InfA KO RHA R | To amplify right homology arm of *infA* downstream region for cloning into pLO3 | TGCAGGTTTAAACAGTCGACTCTAGATCAGGCGGCACCTGGC |
| infA SNP F | To amplify right homology arm of *infA* region with altered nucleotide for cloning into pLO3 | CGTCGAAATCTGAGCATACGCTTCGG |
| infA SNP R | To amplify left homology arm of *infA* region with altered nucleotide for cloning into pLO3 | CGAAGCGTATGCTCAGATTTCGACGCC |
| infA seq F | Confirmation of single and double crossover of pCWS20 and pCWS21 | CTTCACGTCCGAGGATGATGGC |
| infA seq R | Confirmation of single and double crossover of pCWS20 and pCWS21 | CTGATTTTGGCGGGACAAAATGGG |
| infA det F | Confirmation of single and double crossover of pCWS20 and pCWS21 | CAGGATTTTTAAAATCGCAACCACGC |
| infA det R | Confirmation of single and double crossover of pCWS20 and pCWS21 | TGCAGAAACTCAGGACTTGTGGC |

**Additional file 8: Supplementary Table S4.**

**Growth rates for supplementary figures**

| **Figure / Experiment** | **Growth rate h^-1^** |  |
| --- | --- | --- |
|  |  |  |
|  |  |  |
| **Figure S1** |  |  |
|  |  |  |
| Wild type | 0.126±0.011 |  |
| EA | 0.234±0.037 |  |
| EB | 0.274±0.032 |  |
| EC | 0.269±0.026 |  |
|  |  |  |
| **Figure S2** |  |  |
|  |  |  |
| H16 pCWS17 0 mM arabinose | 0.134±0.015 | |
| H16 pCWS17 0.1 mM arabinose | 0.102±0.010 | |
| H16 pCWS17 0.2 mM arabinose | 0.107±0.008 | |
| H16 pCWS17 0.3 mM arabinose | 0.109±0.015 | |
| H16 pCWS15 0 mM arabinose | 0.116±0.015 | |
| H16 pCWS15 0.1 mM arabinose | 0.110±0.006 | |
| H16 pCWS15 0.2 mM arabinose | 0.118±0.016 | |
| H16 pCWS15 0.3 mM arabinose | 0.116±0.013 | |
|  |  |  |

| **Figure S3** | |  |
| --- | --- | --- |
|  | |  |
| Wild type | | 0.051±0.019 |
| *cydA2B2* SNP | | 0.058±0.018 |
| E1 | | 0.046±0.008 |
|  |  |  |
| **Figure S4a** | |  |
|  | |  |
| Wild type | | 0.046±0.018 |
| synE | | 0.140±0.016 |
| *cydA2B2* SNP | | 0.043±0.014 |
| *cydA2B2* Evo1 | | 0.113±0.038 |
| *cydA2B2* Evo2 | | 0.103±0.036 |
| *cydA2B2* Evo3 | | 0.097±0.018 |
|  | |  |
| **Figure S4b** | |  |
|  | |  |
| Wild type | | 0.118±0.045 |
| synE | | 0.127±0.034 |
| *cydA2B2* SNP | | 0.235±0.034 |
| *cydA2B2* Evo1 | | 0.251±0.024 |
| *cydA2B2* Evo2 | | 0.246±0.011 |
| *cydA2B2* Evo3 | | 0.243±0.029 |
|  | |  |
